# Are Cell Junctions Implicated in the Regulation of Vitellogenin Uptake? Insights from an RNAseq-Based Study in Eel, *Anguilla australis*

**DOI:** 10.3390/cells11030550

**Published:** 2022-02-04

**Authors:** Lucila Babio, P. Mark Lokman, Erin L. Damsteegt, Ludovic Dutoit

**Affiliations:** Department of Zoology, University of Otago, 340 Great King Street, P.O. Box 56, Dunedin 9054, New Zealand; mark.lokman@otago.ac.nz (P.M.L.); erin.damsteegt@otago.ac.nz (E.L.D.); ludovic.dutoit@otago.ac.nz (L.D.)

**Keywords:** vitellogenesis, vitellogenin uptake, tight junction, gap junction, vitellogenin receptor, endocytosis

## Abstract

At the onset of puberty, ovarian follicles become competent to incorporate large amounts of vitellogenin (Vtg). Using an RNAseq-based approach, transcriptomes from pre-vitellogenic (PV) and early vitellogenic (EV) ovaries from wild-caught eel, *Anguilla australis*, were compared to investigate the expression of specific genes encoding cell junction proteins that could be involved in regulating Vtg uptake. Partial support was found for the mechanical barrier hypothesis proposing that the access of Vtg to the oolemma is restricted by a tight junction (TJ) network within the granulosa cell layer, which changes between the PV and EV stage. Among 25 genes encoding TJ-constituting proteins, five were down-regulated and two were up-regulated. A chemical barrier hypothesis stating that gap junctions (GJs) are involved in modulating Vtg uptake was not supported, as only five GJs were found to be expressed in the ovary with no significant changes in expression between stages. Furthermore, the endocytic pathway was found to be up-regulated during the PV-EV transition. Finally, the study showed that gene expression patterns may help identify suitable candidates involved in the regulation of Vtg uptake, and provided novel sequence data for *A. australis*, including putative Vtg receptors corresponding to Lr8 and Lrp13 members of the low-density lipoprotein receptor family.

## 1. Introduction

In order to produce high quality eggs that have the ability to self-sustain the development of viable offspring, female oviparous animals need to actively deposit nutrients into the developing oocytes to form the egg yolk: a mass rich in lipids and proteins that will serve as a food source for the future embryo. The developmental period associated with yolk accrual into the oocyte is known as vitellogenesis (vitellogenic growth)—it is characterised by the incorporation of the bulk of the yolk proteins that are obtained through the uptake of very high-density lipoproteins, so-called vitellogenins (Vtg), from the circulation. During this growth phase, different molecular events occurring within the ovarian follicle can affect Vtg uptake. The Vtg receptor (Vtgr)—a member of the low-density lipoprotein receptor (LDLR) supergene family—is probably the most distinct factor. However, extensive evidence in many teleost species indicates that the *vtgr* gene is already expressed and translated at early stages of development [1,2,3], and its transcription either decreases [3,4] or remains constant during subsequent oocyte growth [5]. Indeed, the fact that pre-vitellogenic (PV) follicles are not competent to internalise Vtg in vivo nor in vitro [6], even though the receptors are available [1], prompts the notion that other elements must be involved in activating and supporting the oocyte’s ability to accumulate great quantities of Vtg [6].

Following estrogen-induced synthesis in the liver, Vtg is transported to the ovary via the bloodstream where it exits the capillaries surrounding the theca cells. Once there, it must make its way through the intercellular spaces among granulosa cells to reach the oocyte membrane [7]. Indeed, open channels between the granulosa cells, associated with oocyte developmental stage and size, were shown to provide access for exogenous proteins to the oolemma in various oviparous animals [8], including *Coturnix japonica* [9], *Fundulus heteroclitus* [10], and *Xenopus laevis* [11,12]. Thus, the structural changes that the granulosa cell layer undergoes during oogenesis may well represent the first barrier to affect the accessibility of Vtg to the oocyte. In this context, tight junctions (TJs) create a paracellular barrier to selectively modulate the transport of molecules and water between neighbouring cells [13]. TJs are, for example, known to effectively regulate the epithelial permeability and paracellular transport in many tissues from teleost fish [14], namely gill [15], blood-brain barrier [16], and blood-testis barrier [17]. Furthermore, data from the zebrafish (*Danio rerio*) support a role for TJs in follicle development in view of changes in gene expression of cell junction proteins during oogenesis and their response to endocrine regulation [18]. Hence, a stage-specific TJ network could generate a more “compact” or “leaky” tissue, as such regulating the intercellular space between the granulosa cell layer and restricting or allowing Vtg to reach the oocyte membrane, respectively.

Other structural changes pertaining to cell–cell interactions during follicular development may also be linked to the regulation of Vtg accumulation. Notably distinctive is the appearance of interdigitating projections of microvilli from both granulosa cells and the oocyte, and the assembly of gap junctions (GJs) that connect them, occurring before and during vitellogenic growth (see [19] and references therein). Additionally, the subsequent retraction of the granulosa microvilli during oocyte maturation, (after termination of Vtg uptake and completion of oocyte growth) is well-documented ([19] and references therein). Due to this strong relationship between the granulosa cells and the oocyte during vitellogenic growth, cell junctions involved in cell–cell transport and communication, i.e., GJs, conceivably also play a role in stimulating and maintaining Vtg accrual. Indeed, gap junctional intercellular communication is required for uptake of Vtg in *X. laevis* oocytes [20], associated with the sharing of signalling molecules such as cyclic adenosine monophosphate (cAMP) [21].

In order to investigate the involvement of cell junction proteins in the regulation of Vtg uptake, the following hypotheses were tested: (*i*) a mechanical barrier hypothesis, in which Vtg uptake is regulated by a network of TJs acting as a physical barrier restricting Vtg accessibility to the oolemma, and (*ii*) a chemical barrier hypothesis, in which cell junctions involved in cell–cell transport act as a chemical barrier of an external signalling molecule required for Vtg uptake. Consequently, a transcriptomic approach was used to compare the expression of genes encoding cell junction-constituting proteins between ovaries in the PV stage (previous to Vtg incorporation) and the early vitellogenic stage (EV stage, right after Vtg uptake has started). Additionally, the expression of genes likely involved in the molecular pathway that dictates the fate of Vtg after reaching the oolemma was examined. This included the need to identify the sequences corresponding to Vtgr candidates in the species studied, the New Zealand shortfinned eel, *Anguilla australis* [22]. Like other temperate anguillid eels, this species spends most of its life in freshwater systems until it starts a long oceanic migration towards its breeding grounds. Accordingly, both immature eels and early maturing, migratory eels can be captured from the wild [23], representing the PV and the EV ovarian stages, respectively. Although artificial reproduction of eels has been achieved by applying intense hormonal treatments [24,25], egg hatchability and larval survival rates remain a challenge for a sustainable production at commercial level, which still relies heavily on natural stocks. Elucidation of important mechanisms that assure the production of high-quality eggs, notably Vtg uptake, may help improve freshwater eels’ artificial reproduction and conservation.

## 2. Materials and Methods

### 2.1. Animals and Sample Collection

The study was approved by the University of Otago Animal Ethics Committee and was conducted in accordance with the guidelines of the Australian & New Zealand Council for the Care of Animals in Research and Teaching. Wild shortfinned eels were caught from Lake Ellesmere (South Island, New Zealand; year 2019) using fyke nets. Based on morphological features [26], PV and EV eels (n = 6 per stage) were identified, euthanised with an overdose of benzocaine (0.3 g/L, Sigma-Aldrich, Merck KGaA, Darmstadt, Germany), and dissected at a local makeshift field station. Total body weight was measured and after dissection, ovary and liver were weighed to calculate somatic indices: gonadosomatic index (GSI) and hepatosomatic index (HSI), respectively. Ovarian fragments were fixed in 4% paraformaldehyde for histological analysis (oocyte diameter measurement and confirmation of gonadal stage, Section 2.2), and flash frozen for RNA extraction and next-generation sequencing (Section 2.3).

### 2.2. Histological Analysis

After fixation, ovarian fragments were dehydrated and embedded in Technovit 7100 resin (Heraeus Kulzer GmbH and Co., Hanau, Germany) following the manufacturers’ instructions. Sections were cut at 2 μm using a Reichert-Jung microtome (Model 2050, Cambridge Instruments GmbH, Nussloch, Germany) and stained with Polychrome I (11.3% methylene blue and 0.2% azure II) and Polychrome II (0.2% basic fuchsin) stains. Microscope images were captured with an Olympus camera SC100 and an Olympus adaptor U-TVO.5XC-3 attached to an Olympus microscope BX51 (Olympus Corporation, Tokyo, Japan). Oocyte diameter (OD) was calculated from captured images using ImageJ v1.52a (National Institutes of Health, Bethesda, MD, USA). Oocytes with a visible nucleus were measured according to Lokman et al. [27], and data from the 50% highest-ranked oocytes were retained to calculate the average OD, and to eliminate those oocytes that may have been sectioned off-center.

### 2.3. Total RNA Extraction, cDNA Library Preparation, and High-Throughput Sequencing

Ovarian total RNA was extracted using NucleoSpin RNA Kit (Macherey–Nagel GmbH & Co. KG, Düren, Germany) following manufacturer’s instructions. The total RNA concentration was measured with the Qubit RNA Assay Kit (Thermo Fisher Scientific Inc., Waltham, MA, USA). The relative purity level was determined using a DeNovix spectrophotometer DS-11 (DeNovix Inc., Wilmington, DE, USA), which gave a 260/280 nm absorbance ratio of about 2. The quality and integrity of total RNA was further analysed using an Agilent 5300 Fragment Analyzer (Otago Genomics Services, University of Otago, Dunedin, New Zealand) obtaining the following RNA integrity numbers (RIN): 7.1 ± 1.5 (PV stage, n = 6), and 10 (EV stage, n = 6).

The extracted total RNA was sent frozen using an Ultra-freeze MAX Bio-bottle transport system (Bio-bottle Ltd., Auckland, New Zealand) to the sequencing contractor (Beijing Genomics Institute, BGI, Hong Kong). Once there, a second quality control was performed using an Agilent 2100 Bioanalyzer yielding RIN values of 3.5 ± 0.9, and 9.6 ± 0.2 for the PV and EV stages, respectively (Figure S1, see Section 4).

The strand-specific cDNA library construction (MGIEasy RNA Directional Library Prep Set V2.1, MGI Tech Co. Ltd., a subsidiary of the BGI Group) and subsequent high-throughput sequencing were performed by BGI. Briefly, samples were first enriched for mRNA using oligo-dT selection (Poly(A) mRNA enriched by oligo(dT) beads), and then fragmented and reverse-transcribed. After adaptor ligation and PCR amplification, cDNAs were compacted into DNA nanoballs in order to sequence 100 bp paired-end reads on a DNBseq platform.

### 2.4. Bioinformatics: De Novo Assembly, Annotation, and Differential Expression Analysis

The detailed pipeline for the assembly and differential expression analysis is available at github.com/ldutoit/eelRNA. Briefly, the quality of the data was assessed by FastQC v0.11.9 [28], and the de novo transcriptome assembly was then conducted using Trinity v2.845 [29] specifying each sample’s ovarian developmental stage (PV/EV) in the Trinity samples file. Raw reads were processed using *the align_and_estimate.pl* script distributed with Trinity to align reads using Bowtie v1.2.0 [30,31] before quantifying them at the gene level using RSEM v1.3.2 [32] with SAMtools v1.8 [33]. Candidate coding regions within the assembled transcript sequences were identified using Transdecoder v5.5.0 [34]. The functional annotation was done through Trinotate v3.2.1 [35] combining information from BLASTx and BLASTp [36] against the UniProtKB/SwissProt database [37].

Initial clustering was performed by visualising Euclidean distance between vst-transformed counts (variance stabilising transformation) using the *pheatmap* v1.0.12 [38] and the *DeSeq2* v1.22.2 packages [39] in R v3.5 [40]. Differential expression (DE) analysis was performed using the *edgeR* 3.24.3 [41,42] and the *limma* package v3.38.3 [43]. Genes with small counts were filtered out using the *filterByExpr* function from the *edgeR* package. The *voom* function [44] was then used to transform count data to log_2_ counts per million (logCPM), to normalise counts using quantile normalisation, and to estimate the mean-variance relationship before linear modelling using *lmFit* and *eBayes* functions [45]. A gene ontology (GO) enrichment analysis carried out with Goseq v1.22.0 [46] was used as an exploratory analysis. All genes with an adjusted *p* value after FDR correction (*q* value) of 0.05 [47] were considered. Later, an exhaustive search of target genes was conducted (Section 2.5) where DEGs were identified with a *q* value < 0.05 and a threshold of 2-fold change (2-FC, i.e., −1 ≥ log_2_FC ≥ 1).

### 2.5. Target Genes

Target genes encoding proteins constituting cell junctions, i.e., GJs and TJs (Table 1), as well as genes involved in clathrin-mediated endocytosis, intracellular vesicle trafficking, and Vtg processing (Table S2), were searched on the annotated transcriptome, and their deduced protein sequences were retrieved. Gene nomenclature was based on the corresponding *A. anguilla* top hit of a BLASTp search against the nr protein database from the National Centre for Biotechnology Information (NCBI) [48], except for the Vtgr candidates found. The *A. anguilla* sequences on NCBI correspond to RefSeq GCF_013347855.1, Annotation Release 100 (accessed on 26 August 2021). Then, the target genes were checked for differential expression using the previously established cut-off. Only the genes passing the filtering were used (a higher number of genes encoding cell junctions, i.e., claudins and GJs, were found in the transcriptome with very low expression to be used in DE analysis). When multiple gene IDs were found to be associated with the same functional annotation, only one gene ID was used based on their highest read counts, unless it was confirmed that they correspond to different gene sequences after nucleotide and protein alignments. Additionally, a heatmap of genes involved in the pathway associated with Vtg uptake was visualised using the R package *pheatmap* v1.0.12 [38] after transformation of the counts with the *DeSeq2* v1.26.0 implemented regularised log transformation [39].

The protein sequences corresponding to the Vtgr candidates, deduced from complete open reading frames, were further analysed using the CD-Search tool [49] from the Conserved Domain Database on NCBI [50], and they were subjected to phylogenetic analysis. To construct a phylogenetic tree, amino acid sequences corresponding to members of the LDLR family (low-density lipoprotein receptor 8—LR8—, low-density lipoprotein receptor-related protein 13—Lrp13—, and low-density lipoprotein receptor-related protein 4—LRP4—) from different species were retrieved from NCBI (see species and accession numbers in Figure S2). After alignment of sequences using the ClustalW algorithm [51], the tree was constructed in MEGA v7 [52] using the Maximum Likelihood method based on the JTT matrix-based model [53] applying 1000 bootstrap replicates. A discrete Gamma distribution was used to model evolutionary rate differences among sites (2 categories, +G parameter = 1.7003). The rate variation model allowed for some sites to be evolutionarily invariable ([+I], 8.87% sites). All positions with less than 95% site coverage were eliminated. The LRP4 members were used as out-group.

### 2.6. Statistics

Data from somatic indices and OD were tested for normality and equal variances using the Shapiro–Wilk [54] and Levene’s [55] tests, respectively, and arcsine transformed when necessary (GSI and HSI). Thereafter, the data were subjected to a t-test using InfoStat v.2018 [56] to compare PV and EV eels. All gene sets studied were evaluated under the Fisher’s exact test in R v3.5 [40] to check their enrichment in the DEGs group.

## 3. Results

### 3.1. Characterisation of Ovarian Developmental Stage

**Figure 1:**
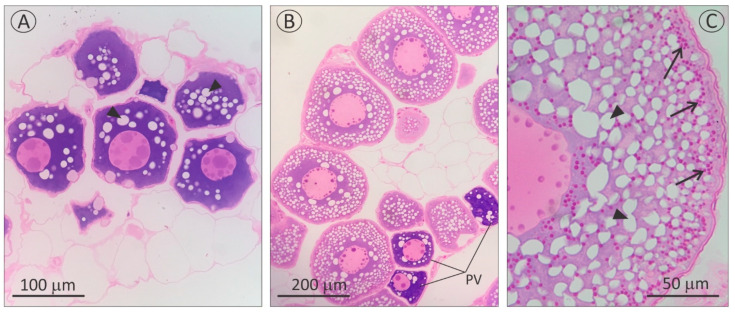
Histological section from pre-vitellogenic (PV: (**A**)) and early vitellogenic (EV: (**B**,**C**)) ovaries from wild-caught *A. australis* (year 2019). A noticeable increase in oocyte size and the presence of yolk proteins (arrows) differentiate the EV stage from the PV stage. EV ovaries normally contain few PV oocytes, as noted in (**B**). Black arrowhead: lipid droplets.

When progressing from the PV to the EV stage, significant increases were seen in the GSI (from 0.89 ± 0.17% to 3.28 ± 0.69%, t = 7, df = 10, *p* value < 0.0001), the HSI (from 0.57 ± 0.04% to 1.04 ± 0.13%, t = 7.11, df = 10, *p* value < 0.0001), and the OD (from 109 ± 6 μm to 271 ± 6 μm, t = 14.56, df = 10, *p* value < 0.0001), respectively. Further histological analysis of ovaries confirmed the developmental stages assigned using morphological traits while sampling. An evident increase in oocyte size due to great accumulation of lipid droplets and yolk proteins (Vtg incorporation) was seen in EV oocytes when compared to PV oocytes, which only showed a few lipid droplets and no signs of Vtg accumulation in their cytoplasm (Figure 1).

### 3.2. De Novo Transcriptome and Downstream Analysis

**Figure 2:**
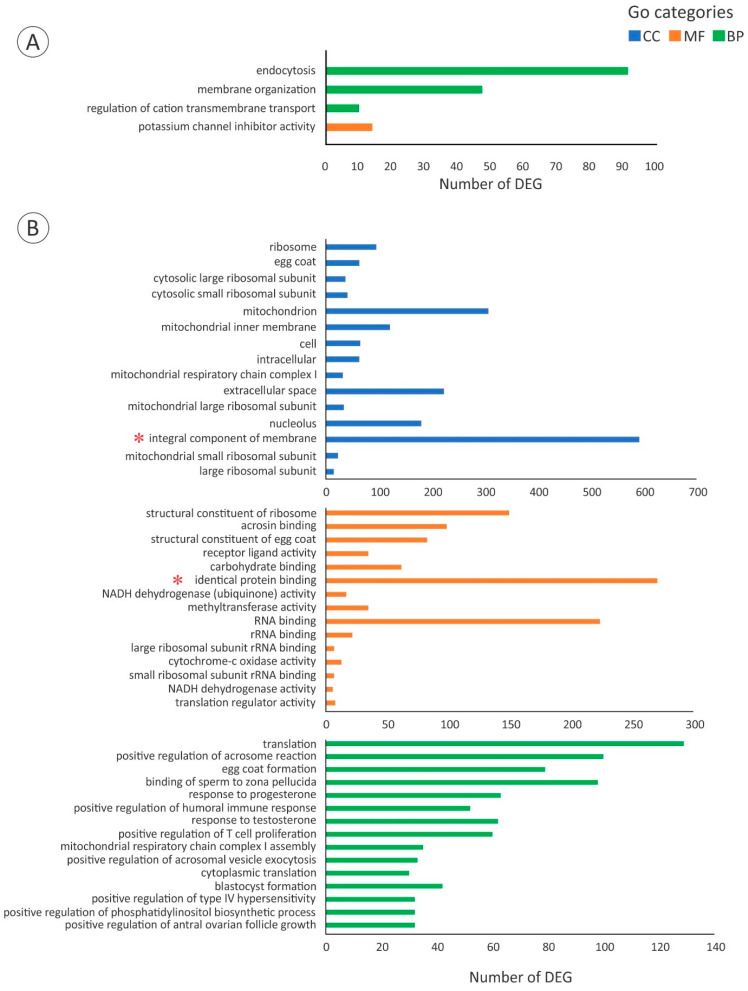
(**A**) All up-regulated GO terms in *A. australis* ovary when progressing from the PV stage to the EV stage. (**B**) Fifteen most down-regulated GO terms from Biological Process (BP), Molecular function (MF), and Cellular Component (CC) categories during the PV-EV transition. The integral component of membrane and identical protein binding terms are accompanied by a red asterisk.

An average of 14.25 million 100 bp paired-end reads per sample were sequenced (min: 12.73, max: 14.65). The de novo assembly generated 282,469 transcripts for 171,620 putative genes, of which 43,975 (25.6%) were functionally annotated. After filtering out lowly expressed genes, 32,447 genes were kept, of which 20,810 (64.1%) were functionally annotated. Initial clustering performed using the Euclidean distance between samples was visualised on a heatmap to assess the variability within the dataset (Figure 2). The samples clustered into two distinct phenotypic groups, PV and EV, indicating that the between-group variance was greater than the within-group variance.

**Figure 3:**
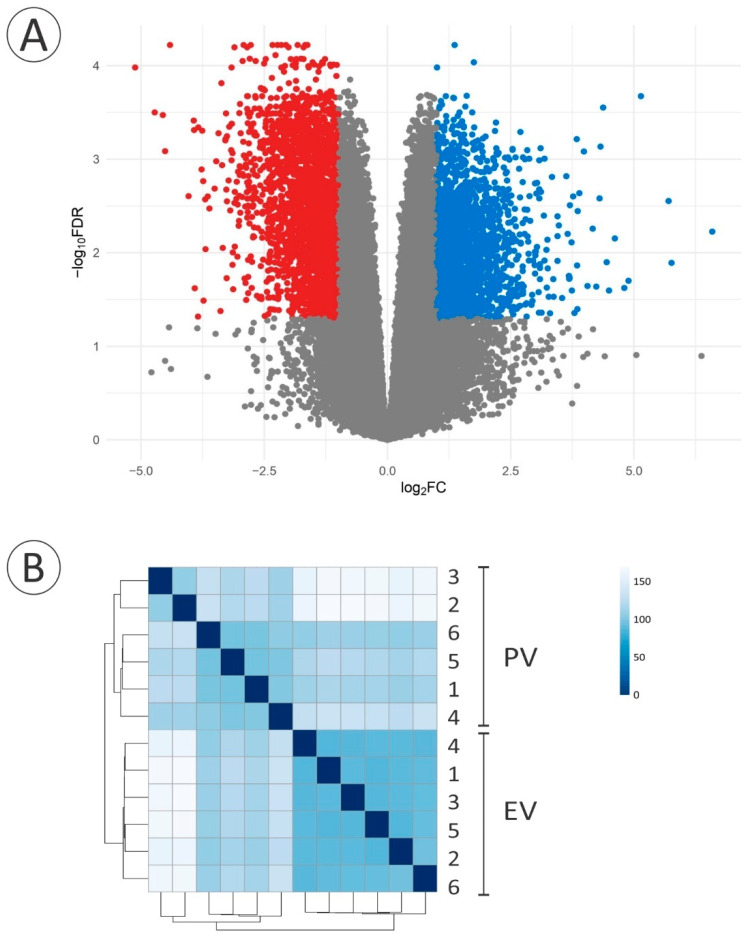
(**A**) Volcano plot displaying significant differentially expressed genes between *A. australis* ovarian tissue in the PV and the EV stage. Under the selected cut-off (*q* value < 0.05; −1 ≥ log_2_FC ≥ 1), a total of 4878 genes were differentially expressed out of 32,447 genes. While 2027 genes were down-regulated (blue dots), 2851 genes were up-regulated (red dots) during the PV-EV transition. (**B**) Heatmap showing the clustering of samples by Euclidean distance. Each sample (numbered 1–6) represents a biological replicate corresponding to the PV or the EV stage.

The GO enrichment analysis showed the up-regulation of three biological processes (BP) and a molecular function (MF) when progressing from the PV to the EV stage. The up-regulated BP terms included endocytosis (GO:0006897, *q* value 7.6 × 10^−6^) and membrane organisation (GO:0061024, *q* value 0.02) (Figure 2, Table S1), representative genes of which are also denoted in Section 3.4. The third BP term (regulation of cation transmembrane transport, GO:1904062, *q* value 0.03), along with the MF term (potassium channel inhibitor activity, GO:0019870, *q* value 0.04), included a group of genes involved in modulating the frequency, rate or extent of cation transmembrane transport corresponding to the serine/threonine protein kinases WNK1, WNK2, and WNK3 (Figure 2, Table S1). Among the most down-regulated GO terms during the PV-EV transition, integral component of membrane (GO:0016021, *q* value 3.3 × 10^−8^, cellular component -CC- category) and identical protein binding (GO:0042802, *q* value 3.4 × 10^−5^, MF category) (Figure 2, Table S1) included genes encoding TJ-constituting proteins denoted in Section 3.3. Finally, 4878 genes were differentially expressed with a *q* value < 0.05 and a threshold of 2-FC; 2851 genes were up-regulated and 2027 genes were down-regulated when progressing from the PV to the EV stage (Figure 3).

### 3.3. Gene Expression of Cell Junctions during the PV-EV Transition

The group of candidate TJ-constituting proteins was not significantly enriched among the DEGs (Fisher’s exact test, *p* value = 0.07). Among the 25 identified genes encoding different types of TJ-constituting proteins, seven were differentially expressed; only two genes were up-regulated during the PV-EV transition (genes encoding claudin-12 and MARVEL domain containing protein 2-like—MarvelD2-like/tricellulin-like—), whereas five genes were down-regulated (genes encoding claudin-like protein ZF-A9, claudin-3-like, MARVEL domain containing protein 3—MarvelD3—, claudin-like protein ZF-A89, and junctional adhesion protein-2A—Jam2a—) (Table 1). In contrast, five claudins, occludin-like, the angulins lipolysis stimulated lipoprotein receptor (Lsr), immunoglobulin-like domain-containing receptor 1 (Ildr1a), and immunoglobulin-like domain-containing receptor 2 (Ildr2), the tight junction proteins ZO-1-like, ZO-2, ZO-2-like, and ZO-3, Jamb-like, and Jam3b were not differentially expressed between stages (Table 1). Furthermore, only five genes encoding GJ proteins were found (gap junction beta-3 protein-like, gap junction beta-4 protein-like, gap junction gamma-1 protein-like, connexin 28.8, and connexin 34.5), all being not differentially expressed between stages (Fisher’s exact test, *p* value = 1) (Table 1).

### 3.4. Expression of Genes Related to Receptor-Mediated Endocytosis Machinery and Vtg Processing during the PV-EV Transition

Two genes encoding putative Vtgrs were found in the *A. australis* de novo transcriptome based on their annotation and sequence similarity to other vertebrate Vtgrs (analysis of conserved domains from deduced protein sequences and alignment with corresponding LDLR family members [57], and phylogenetic tree constructed using LR8 and Lrp13 members from several species along with LRP4 members as the out-group, Figure S2). The gene TRINITY_DN701_c1_g1 was designated as *lr8*, while the gene TRINITY_DN2157_c0_g1 functionally annotated as an LRP4 member was actually designated as *lrp13*.

**Figure 4:**
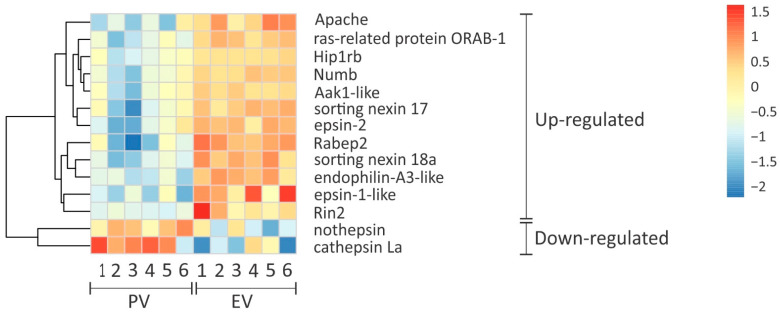
Heatmap of differentially expressed genes involved in clathrin-mediated endocytosis, vesicle trafficking, and proteolysis that could be involved in Vtg uptake pathway in *A. australis*. Ovarian gene expression, represented by colour intensity, is compared between the PV stage and the EV stage. Colour scale gradient from red to blue represents levels of gene expression ranging from large to small, respectively. Up- and down-regulated genes during the PV-EV transition are shown. Each row represents the expression of a gene across all biological replicates from the PV stage or the EV stage (columns).

The group of genes encoding proteins involved in receptor recognition (Vtg specific), clathrin-mediated endocytosis, vesicle trafficking, and Vtg processing, were significantly enriched in the group of DEGs (16 DEGs, 28 not-DEGs: Fisher’s exact test, *p* value < 0.001). Both *lr8* and *lrp13* genes were not differentially expressed during the PV-EV transition (Table S2). Instead, many genes related to clathrin-mediated endocytosis were up-regulated during the PV-EV transition. Genes encoding cytoplasmic adapter proteins involved in clathrin-coated pit formation, such as phosphatidylinositol-binding clathrin assembly protein (Calm/Picalm, Calm/Picalm-like), protein numb homolog (Numb), AP2-interacting clathrin-endocytosis protein (APache), epsin-2, epsin-1-like, huntingtin-interacting protein 1-related protein b (Hip1rb), and AP2-associated protein kinase 1-like (Aak1-like) were up-regulated (Figure 4, Table S2). Yet, the gene encoding the membrane coat protein clathrin heavy chain-1, and genes encoding the adapters phosphatidylinositol-binding clathrin assembly protein (Calm/Picalma, Calm/Picalmb), low-density lipoprotein receptor adapter protein 1-B (a.k.a. Arhb, Arhb-like), disabled homolog-2 (Dab2, Dab2-like), and a subunit from the adaptor protein complex-2 (AP2 subunit alpha-1), were not differentially expressed (Table S2). Additionally, the gene encoding the membrane-bending protein endophilin A3-like was up-regulated (Figure 4), whereas endophilin A3, genes encoding the vesicle budding protein dynamin 2-like, and the protein responsible for vesicle uncoating auxilin were not differentially expressed between stages (Table S2).

With regard to intracellular vesicle trafficking, genes encoding the sorting nexin-17, sorting nexin-18a, ras and Rab interactor-2 (Rin2), Rab GTPase-binding effector protein-2 (Rabep2), and ras-related protein ORAB-1 were up-regulated during the PV-EV transition (Figure 4), while ras-related protein RAB-4A (Rab4a) and ras-related proteins RAB-5A (Rab5aa, Rab5ab), responsible for early endosome trafficking, were not differentially expressed (Table S2). The genes encoding the ras-related protein RAB-35 (Rab35, Rab35b), responsible for recycling endosomes, were not differentially expressed (Table S2). Finally, two genes encoding proteases that could be involved in Vtg proteolysis were down-regulated during the PV-EV transition: cathepsin La and nothepsin (Figure 4), while another six genes encoding cathepsins (D, Z, F, S-like, C, and B) were not differentially expressed between stages (Table S2).

## 4. Discussion

A de novo transcriptome from *A. australis* ovarian tissue was successfully constructed, and an RNAseq approach was used to compare the transcriptomes from PV and EV ovaries. After histological analysis, the sampled groups of wild-caught eels (n = 6 per stage) were a clear representation of these developmental stages, characterising the progression into the early period of vitellogenic growth, when Vtg uptake and accumulation starts to be evident. Great ultrastructural changes occur to the ovarian tissue during development, which are also reflected in its RNA profiling. Normally in teleost fish, ovarian RNA composition changes from a low amount of 18S and 28S rRNA and high amount of 5S rRNA at early stages of development, to higher abundance of 18S and 28S rRNA in more advanced stages [58,59,60,61]. A similar pattern was found in *A. australis* total RNA profiles from both stages, reflecting the differences in the RIN values (Figure S1). The GO enrichment analysis also reflected the differences in transcription between PV and EV stages. While only 3 BP terms (endocytosis GO:0006897, membrane organization GO:0061024, and regulation of cation transmembrane transport GO:1904062) and 1 MF term (potassium channel inhibitory activity GO:0019870) were up-regulated during the PV-EV transition, more than 15 terms were instead down-regulated in each GO category, mainly related to RNA metabolism and egg coat formation.

The RNAseq data was further used to investigate which genes may be involved in promoting competence to actively accumulate Vtg in this species. Indeed, the possible involvement of cell junctions in regulating Vtg uptake was evaluated by testing two hypotheses (Section 4.1 and Section 4.2). Notably, the down-regulated GO terms integral component of membrane (GO:0016021) and identical protein binding (GO:0042802) were found to contain genes encoding TJ-constituting proteins used in hypothesis 1. Similarly, both the endocytosis and membrane organisation GO terms contained genes evaluated further in Section 4.3, where the expression of genes related to receptor-mediated endocytosis machinery and Vtg processing was examined.

### 4.1. Mechanical Barrier Hypothesis and Expression of TJ-Constituting Proteins during the PV-EV Transition

The TJ network consists of a group of transmembrane proteins, including claudins, junctional adhesion proteins (JAMs), angulin proteins (LSR, ILDR1, ILDR2), and MARVEL domain proteins (occludin, MARVELD2 or tricellulin, MARVELD3), that are externally associated with their counterparts on an adjacent cell [62]. Intracellularly, they bind to peripheral membrane proteins (tight junction ZO-1, ZO-2, ZO-3) which in turn make contact with the actin cytoskeleton [63]. The main functions of TJs are to maintain tissue integrity and cell polarity, as well as to modulate the permeability by restricting paracellular transport [13]. Evidently, macromolecules like Vtg are able to diffuse across the granulosa cell layer and reach the oocyte surface during active vitellogenic growth. The results obtained in this study showed that among 25 genes encoding TJ-constituting proteins, five genes were down-regulated compared to the two genes up-regulated during the PV-EV transition.

Between neighbouring cells, specific TJ-constituting proteins tend to localise along bicellular contacts, creating so-called bicellular TJs (bTJs), of which claudins are the major components determining the paracellular permeability [13]. The evident down-regulation in gene expression of TJ-encoding elements during the PV-EV transition in *A. australis* (claudin-like protein ZF-A9, claudin-3-like, MarvelD3, claudin-like protein ZF-A89, and Jam2a) matches the current evidence available from other teleost fish reinforcing the idea that stage-specific changes in TJ-constituting protein abundance may contribute to modulating Vtg paracellular transport. In *D. rerio*, from a total of 20 genes studied (18 claudins and 2 occludins), six claudins were down-regulated from the PV stage to mid-/late-vitellogenic stage [18], whereas in *Gadus morhua*, more than 10 genes involved in the TJ pathway were down-regulated from the PV stage to the EV stage [64]. However, the expression of different types of TJ-constituting proteins varies between the species studied, suggesting that species-specific interactions may be ruling this phenomenon. For instance, through observing a stage-specific down-regulation of occludin, Schuster et al. [65] suggested that in avian follicles TJs prevent the access of Vtg to the oocyte surface during early development. Yet, in the present study, the occludin-like gene was not differentially expressed during the PV-EV transition, indicating it may not play a key role during these stages, or it may carry out other functions in *A. australis* ovarian tissue.

In mammals, a mechanism through which an epithelium can allow the passage of macromolecules without compromising tissue integrity has been proposed. At tricellular contacts, macromolecules are able to diffuse through a central tube formed by bTJs and tricellular TJs (tTJs) [13,66,67]. Tricellulin and angulins are mainly localised at these contact points and may be responsible for sealing this paracellular pathway [66,68,69]. Furthermore, a similar mechanism appears to control paracellular transport through the follicle epithelium during oogenesis in insects (*Drosophila* sp.) [70], indicating it could be well-conserved across distant taxa. Based on this, we were expecting PV ovaries to highly express tTJ-constituting proteins in order to establish a barrier for macromolecules. However, the genes encoding angulins were not differentially expressed between stages, and unexpectedly, a gene encoding a tricellulin-like protein (MarvelD2-like) was up-regulated in EV ovaries. Then, is it possible that the down-regulation of TJ-constituting proteins is not the only mechanism driving a change in the TJ network promoting permeability? Indeed, there are contradicting findings on how tricellulin expression may affect the macromolecular pathway. Although there is evidence indicating that at low tricellulin expression a pathway for macromolecules is formed in mammalian cells [71], when studying an EpH4 mouse mammary epithelial cell line and comparing wild-type versus tricellulin knockdown cells (by RNA interference), Ikenouchi et al. [66] discovered that the flux of 250 Kda FITC-dextran did not differ between cell types. Accordingly, the increase in expression of the tricellulin-like coding gene in *A. australis* EV ovary may not be indicative of a decrease in macromolecule flux, as active Vtg accumulation has already started at this stage.

Although genes encoding TJ-constituting proteins were not enriched in the DE group, and the expression patterns of some TJ-encoding proteins were not as expected (between up-regulation and non-differential expression, i.e., tTJs, occludin), the hypothesis should not be rejected. Instead, alternative ways to test it should be explored aiming to elucidate important questions. What is the main force driving tissue permeability? Is it the abundance of TJ-constituting proteins or the interactions between them which creates a tighter epithelium? Could changes in specific TJ-constituting proteins modify the TJ network? Although each TJ-constituting protein may exhibit a unique function, it is likely that the combination of different proteins is what determines the final permeability of an epithelium. Therefore, the differential expression patterns of specific TJ-constituting proteins between the PV stage and the EV stage (including both up- and down-regulations) may create distinct TJ interactions, which in turn could be affecting the macromolecular flux.

Finally, there were putative paralogs (Marvel domain containing proteins and claudin ZF-A89) showing different patterns of expression in terms of regulation (differential expression between stages) and level of expression (number of reads). Intuitively, those genes contributing with a higher number of reads will be the ones contributing more to the pool of translated proteins. However, the gene expression of follicle cells could be diluted or masked by oocyte-specific transcripts, creating difficulties when interpreting the data. Therefore, the localisation of the expressed genes and their function at the protein level is warranted to elucidate their contribution to the TJ-network during follicle development. Indeed, further information will be needed to fully support the idea that a change in the TJ network may facilitate the accessibility of Vtg to the oocyte surface, in turn modulating Vtg uptake. Targeted studies using qPCR, in situ hybridization, knock down or knock out techniques, and immunohistochemistry would provide valuable data to continue testing this hypothesis.

### 4.2. Chemical Barrier Hypothesis and Expression of GJ Proteins during the PV-EV Transition

During oogenesis, the intercommunication between the oocyte and the granulosa cells is crucial for follicle development [72]. The exchange of information can be done through GJ coupling to establish the formation of channels between neighbouring cells, maintaining a rapid and direct communication to synchronise metabolic activities [73]. In teleost fish, the temporal correlation between the appearance of GJs and the onset of vitellogenic growth suggests the association between the two phenomena [74]. Using *X. laevis* as a model, Monaco et al. [20] concluded that direct GJ communication is required for Vtg uptake as it may be involved in transmitting signalling molecules that may modulate such uptake. Luque et al. [21] subsequently demonstrated that cAMP was the messenger that was transmitted through GJs, regulating the onset and ongoing endocytosis of Vtg in *X. laevis.* Afterwards, Serrano et al. [75] showed that vitellogenic growth in *X. laevis* is actively regulated by an increase in the intracellular levels of Ca^2+^, presumably from the cAMP-induced activation of the IP_3_/ Ca^2+^ pathway. Along with this information, extracellular Ca^2+^ is also known to be required for Vtg receptor binding and subsequent uptake in *Xenopus* oocytes [76,77], suggesting that both intracellular and extracellular levels of Ca^2+^ may be implicated in modulating vitellogenic growth.

Even though the predicted scenario for *A. australis*, in which genes encoding GJ proteins are up-regulated during the PV-EV transition, did not occur, a role for Ca^2+^ signalling during vitellogenic growth in *A. australis* is possible. This notion is supported by the up-regulation of the gene coding for claudin-12 and of the cation transmembrane transport pathway (GO:1904062) in the EV stage. Indeed, both claudin-12 and WNK3, a member of the serine-threonine protein kinases group of genes which were enriched in the biological process GO:1904062, are implicated in Ca^2+^ transport. While the former has been implicated in forming channels for Ca^2+^ transport in mammalian intestinal epithelia [78,79], the latter plays a role as a positive regulator of the transcellular Ca^2+^ transport pathway (human gene expressed in *X. laevis* oocytes [80]). Lastly, both extracellular and intracellular Ca^2+^ are also required for TJ integrity and functioning [81], which further reinforces the possibility that Ca^2+^ may regulate Vtg uptake. Whether these proteins perform the same function in teleost fish needs to be confirmed; however, our current data seem to support the implication of cell junctions and calcium ions in regulation of Vtg uptake. Indeed, a deeper understanding on GJ functioning in PV and EV follicles is still needed to extract comprehensive conclusions.

### 4.3. Expression of Genes Related to Receptor-Mediated Endocytosis Machinery and Vtg Processing during the PV-EV Transition

Following receptor recognition by specific members of the LDLR family, Vtg is incorporated into the oocytes through clathrin-mediated endocytosis [7]. Once inside the oocyte, Vtg is processed into yolk proteins by proteolysis, and the receptor is recycled back to the membrane surface [7]. Consequently, our RNAseq analysis was also used to examine the expression of genes related to Vtg recognition, the molecular machinery of clathrin-mediated endocytosis and vesicle trafficking, and Vtg proteolysis.

Two genes encoding putative Vtgrs were found in the *A. australis* ovarian transcriptome. The deduced proteins from both genes clustered into distinct groups corresponding to Lr8 and Lrp13 members from the LDLR family. Both members have been confirmed to bind Vtg in perciform and salmonid species, i.e., white perch, *Morone americana* [3], rainbow trout, *O. mykiss* [82] and cutthroat trout, *O. clarki* [83]. Therefore, they were both considered in this study as putative Vtgrs. In terms of expression, no difference was found between the PV and EV stages for both genes in *A. australis*. Similar findings for *A. australis* have been previously found for *lr8* expression by Damsteegt et al. [84] and Jéhannet et al. [85], and in *A. anguilla* by Morini et al. [5], reinforcing the idea that expression of putative Vtgr does not seem to be a limiting factor for Vtg uptake.

Of note, Opresko & Wiley [86] discovered that under hormone stimulation, enhanced uptake of Vtg in *Xenopus* oocytes was not due to an increase in Vtgr abundance at the oocyte surface, but to an increase of its internalisation rate. Accordingly, other processes downstream of the endocytic pathway of Vtg (clathrin-mediated endocytosis, vesicle trafficking, Vtg proteolysis) may be involved in the regulation of Vtg accumulation. Sure enough, endocytosis (GO:0006897) and membrane organisation (GO:0061024), both containing genes that actively participate in clathrin-mediated endocytosis, were biological processes found to be up-regulated during the PV-EV transition. Even though clathrin-mediated endocytosis and its associated machinery involved in vesicle trafficking are not exclusive to Vtg uptake, and processes like lipid droplet and cortical alveoli formation might overlap, the active accumulation of Vtg can be considered as the main process happening in the ovary during the PV-EV transition. Knowledge of the function of these specific proteins will be necessary to better understand their contribution to Vtg uptake as they may participate in multiple pathways.

Interestingly, the adaptor protein Arhb, which is known to be required for efficient endocytosis of LDLR family members [87], including that of Vtg in *X. laevis* [88,89], was not differentially expressed between stages, indicating it may not play an essential role during vitellogenic growth in *A. australis*. However, the up-regulation of endophilin A3-like and the near up-regulation of endophilin-A3 (log_2_FC −0.94) in EV ovaries of *A. australis* confirms the pattern found in chicken oocytes. Indeed, Hirayama et al. [90] showed compelling evidence suggesting that in chicken, endophilin A3 is an important component of clathrin-mediated endocytosis during oocyte growth. Finally, other elements from the endocytic machinery (Calm, Numb, sorting nexin 17, etc.) showed differential expression patterns during the PV-EV transition in *A. australis*, differing to what was found in the swordfish *Xiphias gladius* when comparing immature and matured ovaries [91]. While differential expression patterns were also found in *X. gladius*, the DEGs related to the endocytic pathway differed between species.

After endocytosis, Vtg is proteolytically cleaved by specific proteases, i.e., cathepsins. Most of the cathepsins found in the transcriptome were not differentially expressed between PV and EV stages; except for the down-regulation in EV ovaries of cathepsin L1 and nothepsin, the latter likely to be a paralog of cathepsin D [92]. Finally, the early expression of genes encoding these proteases agrees with previous data suggesting that cathepsins may be regulated post-transcriptionally, leading to difficulties when trying to use expression data to explain activity [93,94,95,96]. This issue could be problematic especially in the light of our finding that translation-related GO terms have been down-regulated during the PV-EV transition (MF translation regulator activity GO:0045182, BP cytoplasmic translation GO:0002181, and BP translation GO:0006412). Indeed, additional data on gene and protein expression is needed to further support our findings.

## 5. Conclusions

The assembly of a de novo ovarian transcriptome of *A. australis*, followed by an RNAseq-based study comparing PV and EV ovaries, enabled us to obtain novel gene information from candidates that could be involved in Vtg uptake regulation. Two hypotheses that both implicate cell junctions in Vtg uptake were tested. The first hypothesis—the mechanical barrier hypothesis—was supported on the basis of the differential expression of genes encoding TJ-constituting proteins during the PV-EV transition, suggesting a role for modulating Vtg access to the oocyte surface. No compelling support for the second hypothesis—the chemical barrier hypothesis—was obtained. However, genes encoding clathrin-mediated endocytosis and vesicle trafficking elements were also clearly up-regulated during the PV-EV transition, indicating they, too, may be important factors modulating Vtg accumulation once the protein reaches the oolemma. Lastly, two genes encoding putative Vtgrs were found in *A. australis*, representing Lr8 (including Lr8+/- variants) and Lrp13 members of the LDLR supergene family. Comparing transcriptomes from ovarian tissue at different developmental stages remains a challenge when trying to understand physiological pathways due to different cell types contributing to changing RNA profiles. In addition, the absence of a reliable reference genome plus the known retention of paralogues in teleost fish increases the complexity of next generation sequencing data analysis. However, studies based on comparative analysis of transcript abundances may provide valuable insight into the molecular events and physiological pathways underlying oocyte growth, which can be further investigated in future studies. In conclusion, combination of these data, along with protein expression, functioning, and localisation, is warranted to further unravel these pathways.

## Figures and Tables

**Table 1:** Expression of genes encoding cell junctions during progression from the PV stage to the EV stage in *A. australis* ovarian tissue. Genes encoding tight junction proteins as well as gap junction (gj) proteins are shown. Both differentially and not differentially expressed genes are included, along with their respective *q* values and log_2_FC. Fold change in expression is represented by log_2_FC and it is relative to the PV stage. A positive log_2_FC indicates up-regulation in the PV stage and down-regulation in the EV stage, while a negative log_2_FC means up-regulation in the EV stage and down-regulation in the PV stage. Significant DEGs were considered when showing *q* value < 0.05 and −1 ≥ log_2_FC ≥ 1, representing a 2-FC. Gene names are based on the *A. anguilla* top hit (*A. anguilla* genome, NCBI RefSeq: GCF_013347855.1, Annotation Release 100) from the BLASTp search of deduced protein sequences retrieved from the transcriptome. The read counts (mean ± SEM) are shown for each stage (n = 6 per stage).

Down-Regulated Genes during the PV-EV Transition						
Gene ID TRINITY_	Annotation	*A. anguilla* BLASTp Hit/Gene Associated	Log_2_FC	*q* Value	PV Reads ± SEM	EV Reads ± SEM
DN13517_c0_g1	claudin-14 [*H. sapiens*]	claudin-like protein ZF-A9 [XP_035248997.1]/*cldng*	2.24	0.02	45.5 ± 12.1	14.7 ± 3.8
DN36018_c0_g1	claudin-3 [*R. norvegicus*]	claudin-3-like [XP_035290832.1]/LOC118236501	1.70	0.02	20.8 ± 4.7	9.8 ± 2.1
DN7995_c0_g1	MARVELD3 [*H. sapiens*]	MarvelD3 [XP_035251135.1]/*marveld3*	1.22	<0.01	391.3 ± 20.6	257.5 ± 36.6
DN55532_c0_g1	claudin-like protein ZF-A89 [*D. rerio*]	claudin-like protein ZF-A89 [XP_035288223.1]/LOC118235193	1.13	<0.01	542.7 ± 65.3	356.2 ± 39.2
DN1688_c0_g3	Jam2a [*D. rerio*]	Jam2a [XP_035249352.1]/*jam2a*	1.12	<0.01	252.2 ± 13.5	174.2 ± 8.2
**Up-regulated genes during the PV-EV transition**						
DN10265_c0_g1	claudin-12 [*P. abelii*]	claudin-12 [XP_035258512.1]/*cldn12*	−1.03	<0.01	74.5 ± 14.4	220.3 ± 10.4
DN4344_c0_g2	MARVELD2 [*X. tropicalis*]	MarvelD2-like [XP_035245633.1]/LOC118212123	−1.24	<0.01	92.2 ± 12.4	323.5 ± 29.5
**Not differentially expressed between PV and EV stages**						
DN19581_c0_g1	claudin-7a [*D. rerio*]	claudin-7b-like [XP_035288086.1]/LOC118235138	0.87	0.04	309.3 ± 20.6	266.8 ± 42.6
DN2234_c0_g1	claudin-7a [*D. rerio*]	claudin-7-a [XP_035265529.1]/*cldn7a*	0.13	0.81	2309.4 ± 395.7	2580 ± 490
DN11172_c0_g1	claudin-like protein ZF-A89 [*D. rerio*]	claudin-like protein ZF-A89 [XP_035287488.1]/ LOC118234813	0.37	<0.01	52,673.3 ± 3151.4	30,285.6 ± 1014.1
DN23602_c0_g2	claudin-8 [*M. musculus*]	claudin-8-like [XP_035242392.1]/LOC118210375	−0.43	0.36	10.2 ± 1.9	22.2 ± 3.2
DN19953_c0_g1	claudin-11 [*B. taurus*]	claudin-11a [XP_035278421.1]/*cldn11a*	−0.70	0.08	14.8 ± 2.4	35.8 ± 3.6
DN11142_c0_g1	Jam3b [*D. rerio*]	Jam3b [XP_035242387.1]/*jam3a*	−1.13	0.14	16.8 ± 6.3	55.7 ± 15.9
DN21932_c0_g1	Jam2a [*D. rerio*]	Jamb-like [XP_035266823.1]/LOC118223892	0.59	0.34	10.7 ± 1.9	11.7 ± 2.3
DN6434_c0_g1	occludin [*R. norvegicus*]	occludin-like [XP_035245592.1]/LOC118212103	0.24	0.23	877.5 ± 99.6	958.8 ± 43.3
DN5516_c0_g1	LSR (Angulin-1) [*M. musculus*]	Lsr [XP_035263346.1]/*lsr*	0.28	0.31	623.3 ± 72.6	698.7 ± 74.5
DN4811_c0_g1	ILDR1 (Angulin-2) [*X. laevis*]	Ildr1a [XP_035250686.1]/*ildr1a*	−0.29	0.07	3746.9 ± 339.2	5119.4 ± 317.7
DN9449_c0_g1	ILDR2 (Angulin-3) [*M. musculus*]	Ildr2 [XP_035250586.1]/LOC118214609	−0.51	0.15	11.2 ± 0.9	24.6 ± 1.5
DN198_c0_g1	tight junction ZO-1 [*C. familiaris*]	tight junction ZO-1-like [XP_035273256.1]/LOC118227177	−0.48	0.02	1066.7 ± 132.1	1836.1 ± 99.2
DN5322_c0_g1	tight junction ZO-1 [*C. familiaris*]	tight junction ZO1-like [XP_035251267.1]/LOC118215021	0.04	0.89	84.3 ± 5.8	126.5 ± 10.9
DN7765_c0_g1	tight junction ZO-2 [*M. musculus*]	tight junction ZO-2-like [XP_035292052.1]/LOC118237445	0.87	0.01	460.5 ± 51.0	363.5 ± 48.4
DN5748_c0_g1	tight junction ZO-2 [*H. sapiens*]	tight junction ZO-2a [XP_035247383.1]/*tjp2a*	<−0.01	0.99	1619.3 ± 200.9	1968.6 ± 124.7
DN2057_c0_g1	tight junction ZO-3 [*H. sapiens*]	tight junction ZO-3 [XP_035271907.1]/t*jp3*	−0.40	0.03	2773 ± 270.3	4170.8 ± 221.7
DN2926_c0_g1	MARVELD3 [*H. sapiens*]	MarvelD3 [XP_035270459.1]/si:ch211-191a24.4	0.33	0.42	525.5 ± 82.8	584.505 ± 94.0
DN4344_c0_g1	MARVELD2 [*X. tropicalis*]	MarvelD2b [XP_035235988.1]/*marveld2b*	−0.1	0.50	1209.8 ± 100.3	1624.2 ± 93.8
DN7029_c0_g4	gj gamma 1 protein [*D. rerio*]	gj gamma 1 protein-like [XP_035250527.1]/LOC118214562	−0.31	0.03	787 ± 81	1270.8 ± 47.1
DN998_c2_g1	gj beta-3 protein [*R. norvegicus*]	gj beta-3 protein-like [XP_035290867.1]/LC118236520	−0.15	0.23	5111.3 ± 429.3	6064.8 ± 236.4
DN379_c0_g1	gj beta-4 protein [*R. norvegicus*]	gj beta-4 protein-like [XP_035242057.1]/LOC118210192	0.70	<0.01	180.6 ± 18.3	167.3 ± 13.1
DN15444_c0_g1	gj beta-7 protein [*H. sapiens*]	connexin 28.8 [XP_035277334.1]/*cx28.8*	1.27	0.09	36.8 ± 8.1	24.8 ± 6.3
DN12469_c0_g1	gj 32.7 protein [*M. undulatus*]	connexin 34.5 [XP_035280040.1]/*cx34.5*	−0.88	0.02	41.7 ± 6.1	114.8 ± 15.3

## Data Availability

Raw data were deposited in the Short Read Archive (https://www.ncbi.nlm.nih.gov/sra, accessed on 16 December 2021) under the Bioproject PRJNA785278. The detailed pipeline for the assembly and differential expression analysis is available at github.com/ldutoit/eelRNA.

## Supplementary Materials

The following are available online at https://www.mdpi.com/article/10.3390/cells11030550/s1, Figure S1: Total RNA electropherograms of representative ovarian samples from *Anguilla australis*, Figure S2: Phylogenetic tree of LR8, Lrp13 and LRP4 low-density lipoprotein receptor family members. Table S1: Gene ontology categories up-regulated in ovaries from *A. australis* when progressing from the pre-vitellogenic stage to the early vitellogenic stage, Table S2: Differentially expressed genes related to receptor recognition, receptor-mediated endocytosis, vesicle trafficking, and Vtg processing in *Anguilla australis* ovary.
