# Supplementary material for: Are Cell Junctions Implicated in the Regulation of Vitellogenin Uptake? Insights from an RNAseq-Based Study in Eel, Anguilla australis
Source: Cells. 2022 Feb 4;11(3):550. doi: 10.3390/cells11030550 (PMC8834532; doi:10.3390/cells11030550)
Supplement: Supplementary file 1 [file cells-11-00550-s001.zip › Supplementary Materials - Babio et al., 2022/Suppl Figs - Babio et al. 2022.pdf]

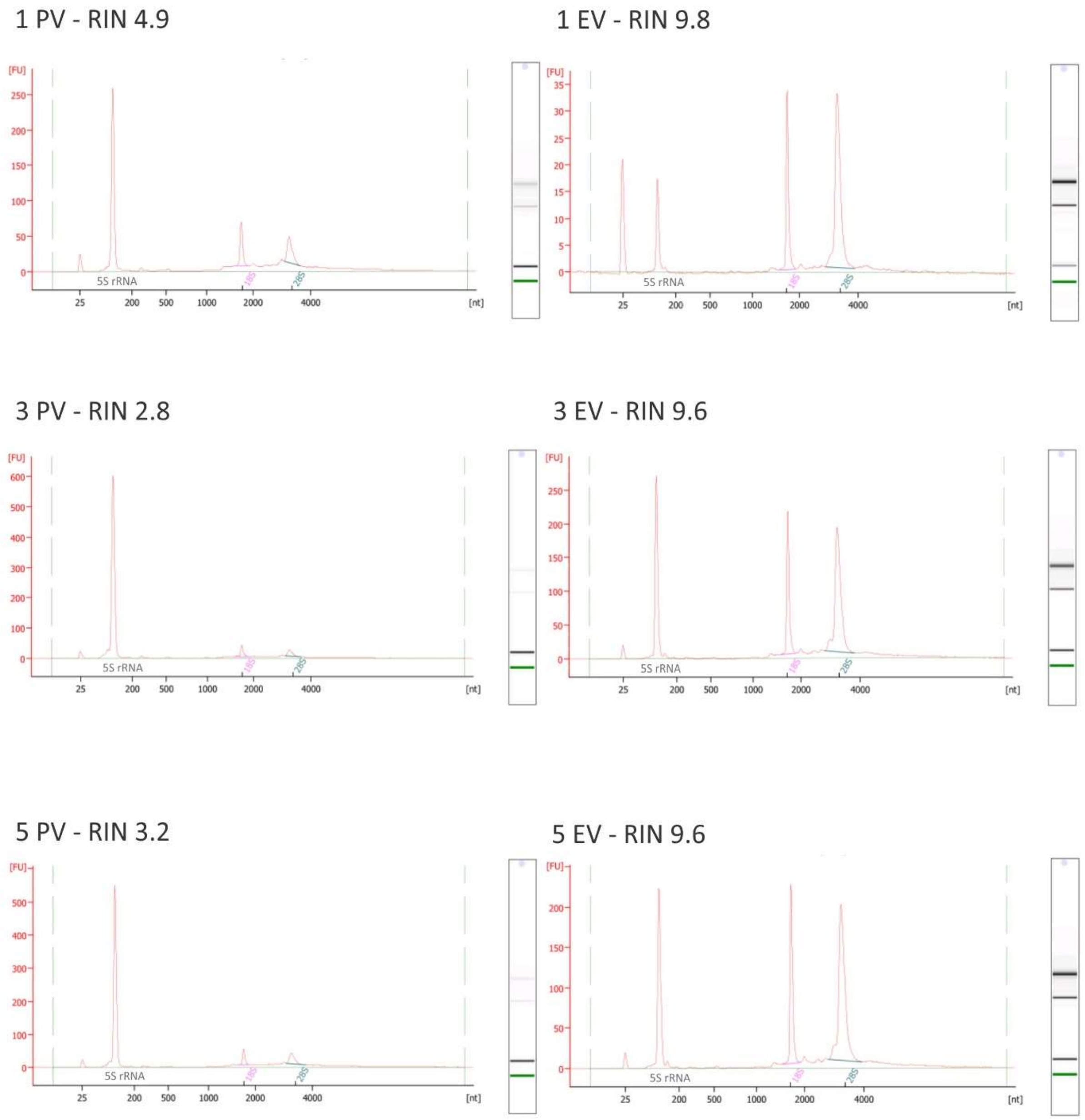

Figure S1. Total RNA electropherograms and gel-like images of 6 representative ovarian samples from *A. australis* in the pre-vitellogenic (PV) and the early vitellogenic (EV) stage that were subjected to high-throughput sequencing. Corresponding 5S, 18S, and 28S ribosomal peaks along with RIN values are shown. All samples presented significant peaks in the 5S rRNA region, with the PV samples showing notably higher peaks than the EV samples. The PV RNA samples also had lower 18S and 28S rRNA peaks in comparison to the EV RNA samples. Even though the RIN values from the PV samples were lower than the EV samples (3.5 ± 0.9 and 9.6 ± 0.2, respectively), no evidence of degradation was detected as the baseline between peaks was smooth.

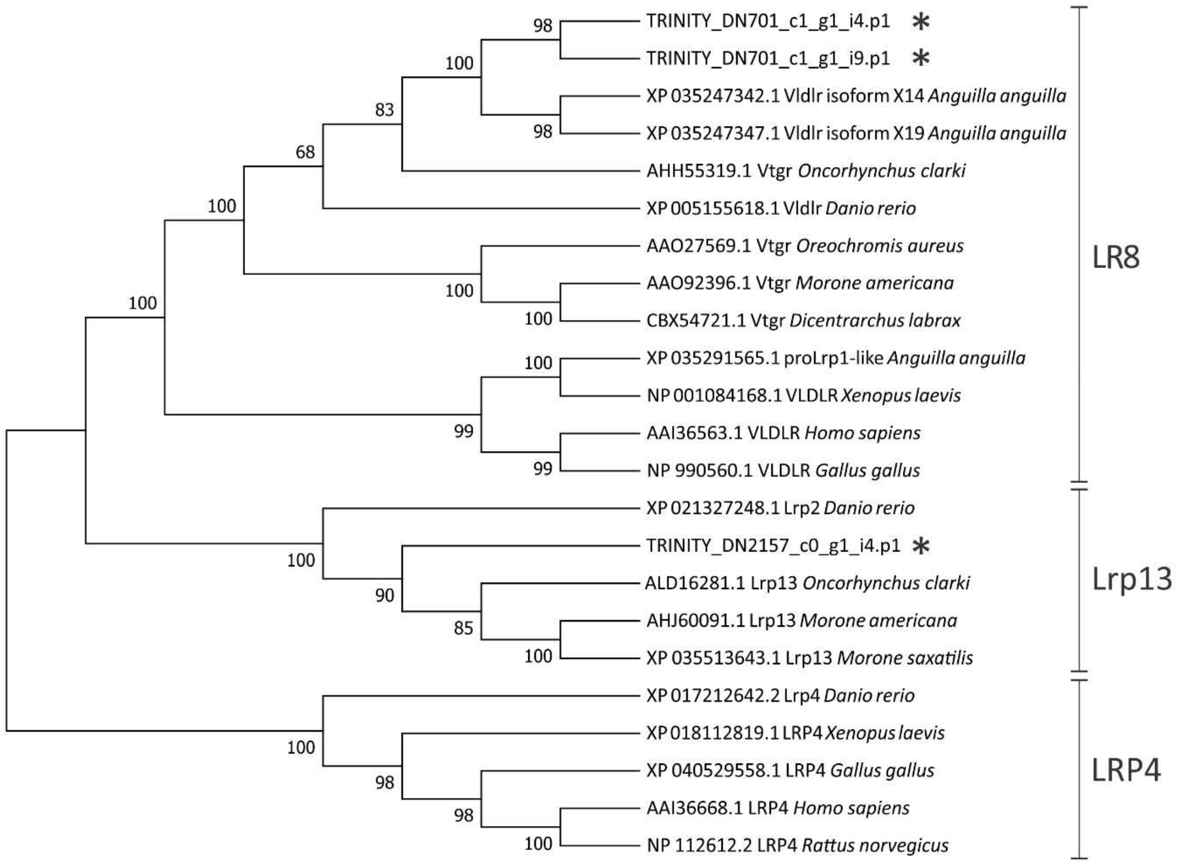

Figure S2. Phylogenetic tree of LR8, Lrp13 and LRP4 low-density lipoprotein receptor family members**.** Deduced protein sequences from putative Vtgr found in *A. australis* (denoted with an asterisk symbol) were grouped according to sequence similarity with lipoprotein receptors from other species (denoted with species name and NCBI accession number). The deduced protein sequences from two *lr8* splice variants—TRINITY_DN701_c1_g1_i4 and TRINITY_DN701_c1_g1_i9 gene isoforms—were detected as Lr8+ and Lr8- variants, referring to the presence or absence of a putative *O*-linked sugar domain, respectively (the same pattern was found in *Oncorhynchus mykiss*: presence or absence of 35 amino acids in same position [126]).LRP4 was used as out-group. Bootstrapping values are shown at tree nodes.

References

126. Prat, F.; Coward, K.; Sumpter, J.P.; Tyler, C.R. Molecular characterization and expression of two ovarian lipoprotein receptors in the rainbow trout, *Oncorhynchus mykiss*. *Biol. Reprod.* **1998**, *58*, pp. 1146–1153, https://doi.org/10.1095/biolreprod58.5.1146
