# Supplementary material for: Are Cell Junctions Implicated in the Regulation of Vitellogenin Uptake? Insights from an RNAseq-Based Study in Eel, Anguilla australis
Source: Cells. 2022 Feb 4;11(3):550. doi: 10.3390/cells11030550 (PMC8834532; doi:10.3390/cells11030550)
Supplement: Supplementary file 1 [file cells-11-00550-s001.zip › Supplementary Materials - Babio et al., 2022/Suppl Tables - Babio et al. 2022.pdf]

Table S1. Gene ontology enrichment analysis showing the genes associated to the three biological processes (BP) and the molecular function (MF) that were up-regulated in ovaries from *A. australis* when progressing from the PV stage to the EV stage. Corresponding GO categories and *q* values are shown.

**Up-regulated GO terms during PV-EV transition**		
**GO term**	**Gene ID**	**Annotation**
BP endocytosis, GO:0006897, *q* value <0.01	TRINITY_DN1152_c1_g1, TRINITY_DN28003_c0_g1, TRINITY_DN13732_c0_g1, TRINITY_DN1500_c0_g1, TRINITY_DN36766_c0_g1, TRINITY_DN9702_c0_g1, TRINITY_DN2157_c0_g1, TRINITY_DN37167_c0_g1, TRINITY_DN105_c0_g1, TRINITY_DN457_c0_g1, TRINITY_DN1565_c1_g1, TRINITY_DN7661_c0_g1, TRINITY_DN11626_c0_g1, TRINITY_DN11818_c0_g1 TRINITY_DN11818_c0_g2, TRINITY_DN2540_c1_g2, TRINITY_DN2540_c1_g3, TRINITY_DN216_c0_g1, TRINITY_DN450_c0_g1, TRINITY_DN2294_c0_g1, TRINITY_DN8930_c0_g1, TRINITY_DN3510_c0_g1, TRINITY_DN69174_c0_g1, TRINITY_DN7762_c1_g1, TRINITY_DN7762_c1_g2, TRINITY_DN8736_c6_g1, TRINITY_DN12183_c0_g1, TRINITY_DN1347_c3_g1, TRINITY_DN1347_c3_g2, TRINITY_DN9803_c0_g1, TRINITY_DN10744_c0_g1, TRINITY_DN28540_c0_g1, TRINITY_DN34737_c0_g1, TRINITY_DN124_c0_g1, TRINITY_DN12586_c0_g1, TRINITY_DN5439_c0_g1, TRINITY_DN1333_c0_g1, TRINITY_DN1339_c0_g2, TRINITY_DN13650_c0_g1, TRINITY_DN42466_c0_g1, TRINITY_DN14594_c0_g1, TRINITY_DN1517_c0_g1, TRINITY_DN15680_c0_g1, TRINITY_DN1599_c0_g1, TRINITY_DN1715_c0_g1, TRINITY_DN2563_c8_g1, TRINITY_DN41011_c0_g1, TRINITY_DN561_c0_g1, TRINITY_DN576_c0_g2, TRINITY_DN17_c4_g1, TRINITY_DN1872_c0_g1, TRINITY_DN191_c0_g1, TRINITY_DN2169_c0_g1, TRINITY_DN22687_c0_g1, TRINITY_DN2311_c0_g1, TRINITY_DN2372_c0_g2, TRINITY_DN2393_c0_g1, TRINITY_DN29887_c1_g1, TRINITY_DN808_c0_g1, TRINITY_DN8136_c0_g1, TRINITY_DN9258_c0_g2, TRINITY_DN3262_c0_g1, TRINITY_DN3262_c0_g2, TRINITY_DN3703_c0_g1, TRINITY_DN408_c0_g1, TRINITY_DN3303_c2_g3, TRINITY_DN350_c0_g1, TRINITY_DN350_c0_g2, TRINITY_DN3766_c0_g1, TRINITY_DN3921_c0_g3, TRINITY_DN4249_c1_g2, TRINITY_DN44_c0_g1, TRINITY_DN4514_c0_g1, TRINITY_DN4531_c0_g1, TRINITY_DN480_c0_g2, TRINITY_DN4898_c4_g1, TRINITY_DN5109_c3_g1, TRINITY_DN8532_c0_g1, TRINITY_DN5212_c0_g1, TRINITY_DN56176_c0_g1, TRINITY_DN58934_c0_g1, TRINITY_DN62163_c0_g1, TRINITY_DN7140_c0_g1, TRINITY_DN7644_c0_g1, TRINITY_DN78598_c0_g2, TRINITY_DN8560_c0_g1, TRINITY_DN916_c0_g1, TRINITY_DN9293_c0_g1, TRINITY_DN9341_c0_g1	LDL receptor related protein 6 [*H. sapiens*], LDL receptor related protein 3 [*H. sapiens*], LDL receptor related protein 3 [*H. sapiens*], LDL receptor [*H. sapiens*], LDL receptor related protein 12 [*M. musculus*], LDL receptor related protein 4 [*R. norvegicus*], growth hormone receptor [*C. familiaris*], epsin-3 [*H. sapiens*], epsin-4 [*M. musculus*], epsin-2 [*H. sapiens*], phosphatidylinositol-binding clathrin assembly protein - CALM [*R. norvegicus*], PI3K-C2-alpha [*H. sapiens*], PI3K-beta [*H. sapiens*], sorting nexin 18a [*M. musculus*], sorting nexin 9 [*H. sapiens*], AP-2 complex subunit alpha-1 [*H. sapiens*], AP2-associated protein kinase 1 - AAK1 [*R. norvegicus*], GAPex-5 [*H. sapiens*], ubiquitin carboxyl-terminal hydrolase 33 [*D. rerio*], ubiquitin carboxyl-terminal hydrolase 20 [*D. rerio*], EH domain-binding protein 1 [*H. sapiens*], GTPase HRas [*G. gallus*], WIP-related protein [*H. sapiens*], myotubularin-related protein 9 [*H. sapiens*], secretory carrier membrane protein 1 [*M. musculus*], oligophrenin-1 [*P. pygmaeus*], RalBP1-interacting protein 2 [*M. musculus*], RIN2 [*M. musculus*], WRCH-1 [*M. musculus*], Rab GTPase-binding effector protein 1 [*M. musculus*], Rab GTPase-binding effector protein 2 [*H. sapiens*], Ras-related protein Rab-34 [*R. norvegicus*], Rab5 GDP/GTP exchange factor [*B. taurus*], Ras-related protein Rab-1A [*R. norvegicus*], target of Myb protein 1 [*H. sapiens*], DENN domain-containing protein 1B [*H. sapiens*], talin-2 [*H. sapiens*], EPS15R [*H. sapiens*], extended synaptotagmin-2 [*M. musculus*], bridging integrator 3 [*M. musculus*], solute carrier family 25 member 36-A [*D. rerio*], unconventional myosin-Ie [*M. musculus*], CD2BP3 [*H. sapiens*], endophilin-A2 [*G. gallus*], endophilin-A3 [*G. gallus*], Intersectin-1 [*M. musculus*], intersectin-2 [*H. sapiens*], MICAL-like protein 1 [*H. sapiens*], chloride transporter ClC-5 [*R. norvegicus*], chloride transporter ClC-4 [*H. sapiens*], RalBP1-interacting protein 1 [*H. sapiens*], E3 ubiquitin-protein ligase mib1 [*D. rerio*], formin-binding protein 1-like [*X. tropicalis*], sortilin [*D. rerio*], Rubicon [*H. sapiens*], WASP family protein member 2 [*B. taurus*], HEAT repeat-containing protein 5B [*H. sapiens*], E3 ubiquitin-protein ligase MARCHF2 [*D. rerio*], synergin gamma [*H. sapiens*], FK506-binding protein 15 [*H. sapiens*], lymphocyte antigen 75 [*H. sapiens*], c-type mannose receptor 2 [*M. musculus*], Rho-related GTP-binding protein RhoV [*R. norvegicus*], tyrosine-protein kinase ABL1 [*M. musculus*], RalBP1-interacting protein 2 [*H. sapiens*], bcl-2-like protein 1 [*S. scrofa*], Rho-related GTP-binding protein RhoV [*B. taurus*], zinc finger FYVE domain-containing protein 9 [*H.*

		sapiens], RalBP1-associated Eps domain-containing protein 1 [*H. sapiens*], actin cytoskeleton-regulatory complex protein PAN1 [Y. lipolytica]
BP membrane organization, GO:0061024, *q* value 0.02	TRINITY_DN105_c0_g1, TRINITY_DN1565_c1_g1, TRINITY_DN7661_c0_g1, TRINITY_DN10842_c0_g1, TRINITY_DN11532_c1_g1, TRINITY_DN12183_c0_g1, TRINITY_DN1347_c3_g1, TRINITY_DN1347_c3_g2, TRINITY_DN9803_c0_g1, TRINITY_DN12695_c0_g1, TRINITY_DN13523_c0_g1, TRINITY_DN14278_c0_g1, TRINITY_DN13675_c0_g1, TRINITY_DN13732_c0_g1, TRINITY_DN14218_c0_g1, TRINITY_DN15006_c0_g1, TRINITY_DN9172_c0_g1, TRINITY_DN2039_c0_g1, TRINITY_DN4848_c0_g1, TRINITY_DN2115_c0_g1, TRINITY_DN2169_c0_g1, TRINITY_DN2169_c1_g1, TRINITY_DN33991_c0_g1, TRINITY_DN216_c0_g1, TRINITY_DN2372_c0_g2, TRINITY_DN2540_c1_g2, TRINITY_DN2540_c1_g3, TRINITY_DN29887_c1_g1, TRINITY_DN3084_c1_g1, TRINITY_DN5647_c0_g1, TRINITY_DN3142_c0_g1, TRINITY_DN3816_c0_g1, TRINITY_DN3262_c0_g1, TRINITY_DN408_c0_g1, TRINITY_DN3510_c0_g1, TRINITY_DN69174_c0_g1, TRINITY_DN3766_c0_g1, TRINITY_DN7644_c0_g1, TRINITY_DN3798_c0_g1, TRINITY_DN5884_c0_g1, TRINITY_DN591_c1_g2, TRINITY_DN6580_c0_g1, TRINITY_DN7762_c1_g1, TRINITY_DN8736_c6_g1, TRINITY_DN8930_c0_g1	epsin-3 [*H. sapiens*], epsin-2 [*H. sapiens*], girdin [*H. sapiens*], transmembrane protein 33 [*H. sapiens*], GTPase-activating protein and VPS9 domain-containing protein 1 [*H. sapiens*], Ras-related protein Ral-A [*H. sapiens*], E3 ubiquitin-protein ligase CBL [*M. musculus*], CI Man-6-P receptor [*H. sapiens*], LDL receptor [*H. sapiens*], auxilin [*H. sapiens*], inositol polyphosphate 5-phosphatase OCRL [*H. sapiens*], synaptojanin-1 [*M. musculus*], calcineurin B homologous protein 1 [*B. taurus*], epidermal growth factor receptor substrate 15-like 1 [*H. sapiens*], epidermal growth factor receptor [*H. sapiens*], phosphatidylinositol 4-phosphate 3-kinase C2 domain-containing subunit alpha [*H. sapiens*], solute carrier family 25 member 36-A [*D. rerio*], phosphatidylinositol-binding clathrin assembly protein - CALM [*R. norvegicus*], CD2BP3 [*H. sapiens*], GTP-binding protein SAR1b [*B. taurus*], disabled homolog 2 - DAB2 [*H. sapiens*], intersectin-1 [*H. sapiens*], AP-2 complex subunit alpha-1 [*H. sapiens*], RalBP1-interacting protein 1 [*H. sapiens*], RalBP1-interacting protein 2 [*M. musculus*], HIP1-related protein [*H. sapiens*], STAM-2 [*H. sapiens*], Arf-GAP domain and FG repeat-containing protein 1 [*B. taurus*], delta(24)-sterol reductase [*H. sapiens*], AP2-associated protein kinase 1 - AAK1 [*R. norvegicus*], sorting nexin 9 [*H. sapiens*]
BP regulation of cation transmembrane transport, GO:1904062, *q* value 0.03	TRINITY_DN11640_c0_g1, TRINITY_DN3519_c1_g1, TRINITY_DN6178_c0_g1, TRINITY_DN16418_c0_g1, TRINITY_DN2343_c0_g2, TRINITY_DN3639_c0_g1, TRINITY_DN51534_c0_g1, TRINITY_DN6347_c0_g1, TRINITY_DN8767_c0_g1, TRINITY_DN9526_c2_g1	serine/threonine-protein kinase WNK3 [*H. sapiens*], serine/threonine-protein kinase WNK1 [*H. sapiens*]
MF potassium channel inhibitor activity, GO:0019870, *q* value 0.04	TRINITY_DN11640_c0_g1, TRINITY_DN3519_c1_g1, TRINITY_DN6178_c0_g1, TRINITY_DN16418_c0_g1, TRINITY_DN2343_c0_g2, TRINITY_DN3639_c0_g1, TRINITY_DN51534_c0_g1, TRINITY_DN6347_c0_g1, TRINITY_DN8767_c0_g1, TRINITY_DN9526_c2_g1, TRINITY_DN5316_c1_g1, TRINITY_DN8328_c0_g2, TRINITY_DN9075_c0_g1, TRINITY_DN99_c0_g1	serine/threonine-protein kinase WNK3 [*H. sapiens*], serine/threonine-protein kinase WNK1 [*H. sapiens*], serine/threonine-protein kinase WNK2 [*H. sapiens*], E3 ubiquitin-protein ligase NEDD4-like [*H. sapiens*], Ras GTPase-activating protein 1 [*H. sapiens*]
**Down-regulated GO terms during PV-EV transition**		

CC integral component of membrane, GO:0016021, *q* value < 0.0001	TRINITY_DN10028_c0_g1, TRINITY_DN10116_c0_g1, TRINITY_DN10150_c0_g2, TRINITY_DN10165_c0_g1, TRINITY_DN10173_c0_g1, TRINITY_DN10251_c0_g1, TRINITY_DN10259_c0_g1, TRINITY_DN10309_c0_g1, TRINITY_DN1047_c0_g1, TRINITY_DN12429_c0_g1, TRINITY_DN1055_c0_g1, TRINITY_DN12160_c0_g1, TRINITY_DN10568_c1_g1, TRINITY_DN10713_c0_g1, TRINITY_DN10726_c0_g1, TRINITY_DN10776_c0_g1, TRINITY_DN10780_c0_g1, TRINITY_DN10884_c0_g1, TRINITY_DN10902_c0_g2, TRINITY_DN10914_c0_g1, TRINITY_DN10964_c0_g1, TRINITY_DN11026_c0_g2, TRINITY_DN1102_c0_g1, TRINITY_DN110984_c0_g1, TRINITY_DN11099_c0_g1, TRINITY_DN11123_c0_g1, TRINITY_DN11172_c0_g1, TRINITY_DN55532_c0_g1, TRINITY_DN11199_c0_g1, TRINITY_DN11287_c0_g1, TRINITY_DN11339_c0_g1, TRINITY_DN11487_c0_g1, TRINITY_DN11517_c0_g1, TRINITY_DN24550_c0_g1, TRINITY_DN28673_c0_g1, TRINITY_DN30537_c0_g1, TRINITY_DN35293_c0_g1, TRINITY_DN38872_c1_g1, TRINITY_DN4130_c0_g1, TRINITY_DN43279_c0_g1, TRINITY_DN44066_c0_g1, TRINITY_DN44906_c0_g1, TRINITY_DN50310_c0_g1, TRINITY_DN50559_c0_g1, TRINITY_DN50767_c0_g1, TRINITY_DN50804_c0_g1, TRINITY_DN51961_c0_g1, TRINITY_DN52492_c0_g1, TRINITY_DN53837_c0_g1, TRINITY_DN58673_c2_g1, TRINITY_DN66094_c0_g1, TRINITY_DN68321_c0_g1, TRINITY_DN71852_c0_g1, TRINITY_DN79096_c1_g1, TRINITY_DN9165_c1_g1, TRINITY_DN11549_c0_g1, TRINITY_DN1156_c0_g1, TRINITY_DN11760_c0_g1, TRINITY_DN11824_c0_g2, TRINITY_DN1182_c0_g2, TRINITY_DN11843_c0_g1, TRINITY_DN11878_c0_g2, TRINITY_DN11883_c0_g2, TRINITY_DN11917_c2_g1, TRINITY_DN1195_c0_g1, TRINITY_DN11970_c0_g2, TRINITY_DN12031_c0_g1, TRINITY_DN12067_c0_g1, TRINITY_DN12067_c0_g2, TRINITY_DN121250_c0_g1, TRINITY_DN12187_c0_g1, TRINITY_DN6184_c0_g1, TRINITY_DN12227_c0_g2, TRINITY_DN12403_c0_g1, TRINITY_DN12417_c0_g1, TRINITY_DN13430_c2_g1, TRINITY_DN142303_c0_g1, TRINITY_DN25123_c3_g1, TRINITY_DN27787_c3_g1, TRINITY_DN30191_c0_g1, TRINITY_DN30893_c0_g1, TRINITY_DN319_c3_g1, TRINITY_DN33430_c0_g1, TRINITY_DN33503_c0_g1, TRINITY_DN34273_c0_g1, TRINITY_DN36440_c0_g1, TRINITY_DN36964_c0_g1, TRINITY_DN36998_c0_g1, TRINITY_DN37115_c0_g1, TRINITY_DN37324_c0_g1, TRINITY_DN37422_c0_g1, TRINITY_DN37519_c0_g1, TRINITY_DN37577_c0_g1, TRINITY_DN37884_c0_g1, TRINITY_DN39077_c1_g1, TRINITY_DN39130_c0_g1, TRINITY_DN39132_c0_g1, TRINITY_DN39201_c0_g1, TRINITY_DN41257_c0_g1, TRINITY_DN41413_c0_g1, TRINITY_DN42527_c0_g1, TRINITY_DN42814_c4_g1, TRINITY_DN42986_c0_g1, TRINITY_DN42994_c0_g1, TRINITY_DN43034_c0_g1, TRINITY_DN43152_c0_g1, TRINITY_DN44389_c2_g1, TRINITY_DN45383_c2_g1, TRINITY_DN45528_c1_g1, TRINITY_DN45750_c0_g1, TRINITY_DN45909_c0_g1, TRINITY_DN47675_c0_g1, TRINITY_DN48090_c1_g1, TRINITY_DN48103_c0_g1, TRINITY_DN50107_c0_g1, TRINITY_DN50361_c0_g1, TRINITY_DN50651_c0_g1, TRINITY_DN50769_c0_g1, TRINITY_DN50771_c0_g1, TRINITY_DN50838_c2_g1, TRINITY_DN51047_c0_g1, TRINITY_DN51167_c0_g1,	Prostaglandin E2 receptor EP3 subtype [*B. taurus*], Cytochrome c oxidase subunit 7C [*S. sciureus*], Sterol regulatory element-binding protein 1 [*H. sapiens*], Solute carrier family 23 member 1 [*M. musculus*], Two pore calcium channel protein 2 [*D. rerio*], Receptor-type tyrosine-protein phosphatase beta [*M. musculus*] Mitochondrial amidoxime-reducing component 1 [*D. rerio*], Mitochondrial sodium/calcium exchanger protein [*M. musculus*], V-type proton ATPase subunit e 1 [*B. taurus*], Matrix-remodeling-associated protein 7 [*M. musculus*], Vacuolar ATPase assembly integral membrane protein vma21 [*D. rerio*], Transmembrane protein 134 [*M. musculus*], Membrane protein MLC1 [*M. musculus*], Plexin-A4 [*D. rerio*], Dual oxidase maturation factor 1 [*H. sapiens*], Cytochrome c oxidase subunit 4 isoform 2 [*T. obesus*], Secretory carrier-associated membrane protein 1 [*S. scrofa*], Transmembrane protein 186 [*D. rerio*], Protein rapunzel [*D. rerio*], Protein Asterix [*G. gallus*], Gamma-secretase subunit PEN-2 [*D. rerio*], Respirasome Complex Assembly Factor 1 [*M. musculus*], Progressive ankylosis protein homolog B [*D. rerio*], Cadherin-1 [*B. taurus*], Claudin-like protein ZF-A89 [*D. rerio*], Glycosaminoglycan xylosylkinase [*D. rerio*], Galanin receptor type 1 [*R. norvegicus*], CDGSH iron-sulfur domain-containing protein 2A [S. salar], Sodium-dependent phosphate transport protein 2A [*H. sapiens*], Zona pellucida sperm-binding protein 4 [*R. norvegicus*], Putative sodium-coupled neutral amino acid transporter 8 [*X. tropicalis*], Transmembrane protein 184B [*M. musculus*], Solute carrier family 43 member 3 [*H. sapiens*], Lamina-associated polypeptide 2 [*M. musculus*], Ig-like V-type domain-containing protein FAM187A [*B. taurus*], XK-related protein 8 [*G. aculeatus*], Sodium/bile acid cotransporter 7 [*H. sapiens*], Nuclear envelope phosphatase-regulatory subunit 1 [*D. rerio*], Endothelin receptor type B [*C. familiaris*], Transmembrane protein 263-B [*X. laevis*], Vit K epoxide reductase complex subunit 1-like prot 1 [*R. norvegicus*], Alg12 [*H. sapiens*], T-cell leukemia translocation-altered gene protein homolog [*D. rerio*], CD63 antigen [*H. sapiens*], Netrin receptor UNC5B [*H. sapiens*], Arylacetamide deacetylase [O. cuniculus], Zona pellucida sperm-binding protein 3 [*G. gallus*], Proteinase-activated receptor 2 [*M. musculus*], Surfeit locus protein 1 [*T. rubripes*], Olfactory receptor 1A1 [*G. gorilla*], N-acetyltransferase 14 [*D. rerio*], Receptor-type tyrosine-protein phosphatase mu [*M. musculus*], Autophagy-related protein 9A [*B. taurus*], Cytokine receptor common subunit gamma [*B. taurus*], Succinate dehydrogenase cytochrome b560 subunit [C. griseus], CUB and sushi domain-containing protein 1 [*H. sapiens*], Voltage-dependent T-type calcium channel subunit alpha-1H [*R. norvegicus*] Aquaporin-4 [*B. taurus*], Transmembrane protein 14C [*M. musculus*], Platelet glycoprotein 4 [*B. taurus*], Band 3 anion exchange protein [*O. mykiss*], Transmembrane protein 158 [*H. sapiens*], XK-related protein 6 [*H. sapiens*], Thyroxine 5-deiodinase [*S. aurata*], Transmembrane protein 69 [*X. tropicalis*], Claudin-14 [*H. sapiens*], Alpha-N-acetylgalactosaminide alpha-2,6-

	TRINITY_DN51378_c0_g1, TRINITY_DN51426_c1_g1, TRINITY_DN51511_c0_g1, TRINITY_DN51570_c1_g1, TRINITY_DN52908_c0_g1, TRINITY_DN53521_c0_g1, TRINITY_DN53733_c0_g1, TRINITY_DN53835_c0_g1, TRINITY_DN55154_c0_g1, TRINITY_DN55635_c0_g1, TRINITY_DN55878_c0_g1, TRINITY_DN56225_c0_g1, TRINITY_DN56493_c0_g1, TRINITY_DN57177_c0_g1, TRINITY_DN60704_c0_g1, TRINITY_DN65173_c1_g1, TRINITY_DN66410_c1_g1, TRINITY_DN66889_c0_g1, TRINITY_DN67146_c0_g1, TRINITY_DN67733_c0_g1, TRINITY_DN70019_c0_g1, TRINITY_DN72305_c0_g1, TRINITY_DN72535_c0_g1, TRINITY_DN76769_c1_g1, TRINITY_DN79328_c0_g1, TRINITY_DN82462_c0_g1, TRINITY_DN83898_c0_g2, TRINITY_DN84101_c0_g1, TRINITY_DN94276_c0_g1, TRINITY_DN12442_c0_g1, TRINITY_DN12442_c1_g1, TRINITY_DN12487_c0_g1, TRINITY_DN12503_c0_g1, TRINITY_DN12662_c0_g1, TRINITY_DN1272_c1_g1, TRINITY_DN1272_c1_g2, TRINITY_DN12779_c0_g1, TRINITY_DN12837_c0_g1, TRINITY_DN12881_c0_g1, TRINITY_DN12916_c0_g2, TRINITY_DN12959_c0_g1, TRINITY_DN13002_c0_g1, TRINITY_DN1303_c0_g1, TRINITY_DN13269_c0_g3, TRINITY_DN133661_c0_g1, TRINITY_DN13375_c0_g1, TRINITY_DN1339_c0_g1, TRINITY_DN134185_c0_g1, TRINITY_DN1349_c0_g1, TRINITY_DN13517_c0_g1, TRINITY_DN13520_c0_g1, TRINITY_DN13594_c0_g1, TRINITY_DN1360_c0_g3, TRINITY_DN13677_c0_g1, TRINITY_DN13692_c0_g1, TRINITY_DN1781_c1_g1, TRINITY_DN13964_c0_g1, TRINITY_DN13990_c0_g1, TRINITY_DN14006_c0_g1, TRINITY_DN1411_c0_g2, TRINITY_DN14161_c0_g1, TRINITY_DN15867_c0_g1, TRINITY_DN1436_c0_g1, TRINITY_DN1436_c0_g2, TRINITY_DN14390_c0_g1, TRINITY_DN143_c2_g1, TRINITY_DN144_c0_g1, TRINITY_DN1461_c0_g1, TRINITY_DN14643_c0_g1, TRINITY_DN14755_c0_g1, TRINITY_DN27532_c0_g1, TRINITY_DN36413_c1_g1, TRINITY_DN38992_c0_g1, TRINITY_DN51325_c0_g2, TRINITY_DN634_c1_g1, TRINITY_DN65137_c0_g1, TRINITY_DN14807_c0_g2, TRINITY_DN1506_c0_g3, TRINITY_DN151_c0_g1, TRINITY_DN15233_c0_g1, TRINITY_DN15884_c0_g2, TRINITY_DN15301_c0_g1, TRINITY_DN15638_c0_g1, TRINITY_DN157_c0_g1, TRINITY_DN15819_c0_g2, TRINITY_DN15894_c0_g1, TRINITY_DN1590_c0_g1, TRINITY_DN15953_c0_g1, TRINITY_DN15958_c0_g3, TRINITY_DN1600_c0_g2, TRINITY_DN16044_c0_g1, TRINITY_DN6533_c0_g2, TRINITY_DN16100_c0_g1, TRINITY_DN16144_c0_g1, TRINITY_DN16423_c1_g1, TRINITY_DN1645_c0_g1, TRINITY_DN16467_c0_g1, TRINITY_DN16584_c0_g1, TRINITY_DN16585_c0_g1, TRINITY_DN1677_c0_g1, TRINITY_DN1678_c0_g1, TRINITY_DN16884_c0_g1, TRINITY_DN16884_c0_g3, TRINITY_DN16931_c0_g1, TRINITY_DN16995_c0_g1, TRINITY_DN5650_c0_g1, TRINITY_DN17012_c0_g1, TRINITY_DN1709_c0_g2, TRINITY_DN1714_c2_g1, TRINITY_DN17220_c0_g1, TRINITY_DN17369_c1_g2, TRINITY_DN17440_c0_g1, TRINITY_DN17596_c0_g1, TRINITY_DN1760_c0_g1, TRINITY_DN17625_c0_g1, TRINITY_DN1763_c0_g1, TRINITY_DN1766_c0_g2, TRINITY_DN17702_c0_g1, TRINITY_DN17739_c0_g1, TRINITY_DN17768_c0_g1, TRINITY_DN1793_c0_g1, TRINITY_DN1793_c0_g2, TRINITY_DN1806_c3_g1,	sialyltransferase 2 [*G. gallus*], Protein shisa-3 homolog [*M. musculus*], Serine incorporator 1 [*M. musculus*], Small integral membrane protein 15 [*D. rerio*], Cytochrome c oxidase subunit 6A [*O. mykiss*], Vesicle transport protein USE1 [*D. rerio*], Syntaxin-5 [*B. taurus*], Vesicle-associated membrane protein-associated protein B [*R. norvegicus*], Dehydrogenase/reductase SDR family member 7B [*D. rerio*], Sarcoplasmic/endoplasmic reticulum calcium ATPase 2 [*F. catus*], Prostaglandin F2 receptor negative regulator [*H. sapiens*], Short transmembrane mitochondrial protein 1 [*D. rerio*], Vesicle transport protein SFT2A [*H. sapiens*], Netrin receptor UNC5C [*M. musculus*], Transient receptor potential cation channel subfamily V member 5 [*R. norvegicus*], Progestin and adipoQ receptor family member 3 [*M. musculus*], Zona pellucida sperm-binding protein 1 [*R. norvegicus*], Calcium uptake protein 3 [*M. musculus*], Protein PF14_0175 [P. falciparum], ATP synthase F(0) complex subunit C3 [*B. taurus*], Solute carrier family 41 member 2 [*M. musculus*], NADH dehydrogenase 1 subunit C2 [*P. troglodytes*], 5,6-dihydroxyindole-2-carboxylic acid oxidase [*B. taurus*], Transmembrane emp24 domain-containing protein 1 [*X. tropicalis*], Transient receptor potential cation channel subfamily M member 3 [*H. sapiens*]<br>Myelin regulatory factor-like protein [*H. sapiens*], C18orf19 homolog B [*D. rerio*], Transmembrane protein 216 [*D. rerio*], Chitin synthase chs-1 [*C. elegans*], Alpha-1,3-galactosyltransferase 2 [*H. sapiens*], UDP-glucuronosyltransferase 2A2 [*M. musculus*], Cytochrome b-c1 complex subunit 9 [*B. taurus*], Alpha-1B adrenergic receptor [*H. sapiens*], NADH dehydrogenase 1 beta subcomplex subunit 4 [*B. taurus*], Small integral membrane protein 8 [*M. musculus*], Apo L domain-containing protein 1 [*H. sapiens*], Long-chain-fatty-acid--CoA ligase 3 [*P. abelii*], Protein C9orf135 [*H. sapiens*], Tetraspanin-18 [*H. sapiens*], Alkylglycerol monooxygenase [*D. rerio*], Prolactin-releasing peptide receptor [*M. musculus*], Receptor-transporting protein 3 [*H. sapiens*], Attractin [*M. musculus*], Cytochrome c oxidase subunit 7A2 [*B. taurus*], V-set and immunoglobulin domain-containing protein 10 [*D. rerio*], UDP-glucuronic acid decarboxylase 1 [*D. rerio*], Organic solute transporter subunit alpha [*D. rerio*], Phosphatidylinositol-glycan biosynthesis class X protein [*H. sapiens*], All-trans retinoic acid-induced differentiation factor [*D. rerio*], Olfactory receptor 52K1 [*H. sapiens*], Integral membrane protein 2B [*G. gallus*], Calcium signal-modulating cyclophilin ligand [*H. sapiens*], VPS10 domain-containing receptor SorCS2 [*H. sapiens*], RING finger protein 121 [*H. sapiens*], Protein CEBPZOS [*H. sapiens*], Type 2 phosphatidylinositol 4,5-bisphosphate 4-phosphatase [*H. sapiens*], Adhesion G protein-coupled receptor A1 [*H. sapiens*], Phosphatidylinositol-3-phosphatase SAC1-B [*D. rerio*], Leukemia inhibitory factor receptor [*M. musculus*], Proton-coupled amino acid transporter 4 [*H. sapiens*], Sodium-dependent dopamine transporter [*R. norvegicus*], Sarcoplasmic/endoplasmic reticulum calcium ATPase 1 [*G. gallus*], Transmembrane emp24 domain-containing protein 10 [*T. rubripes*],

	TRINITY_DN18085_c0_g4, TRINITY_DN18359_c0_g1, TRINITY_DN22391_c0_g3, TRINITY_DN18477_c0_g1, TRINITY_DN3505_c0_g1, TRINITY_DN18513_c0_g1, TRINITY_DN18519_c0_g1, TRINITY_DN18955_c0_g1, TRINITY_DN18658_c0_g1, TRINITY_DN1870_c0_g2, TRINITY_DN1877_c0_g1, TRINITY_DN1878_c0_g1, TRINITY_DN18833_c0_g1, TRINITY_DN18838_c0_g2, TRINITY_DN18904_c0_g3, TRINITY_DN18931_c0_g1, TRINITY_DN19138_c0_g1, TRINITY_DN19251_c0_g1, TRINITY_DN1926_c0_g2, TRINITY_DN19284_c0_g1, TRINITY_DN1928_c1_g1, TRINITY_DN19319_c1_g2, TRINITY_DN19320_c0_g1, TRINITY_DN19330_c0_g1, TRINITY_DN19401_c0_g1, TRINITY_DN19581_c0_g1, TRINITY_DN1960_c0_g1, TRINITY_DN19712_c0_g1, TRINITY_DN19760_c0_g1, TRINITY_DN1982_c0_g1, TRINITY_DN1983_c0_g1, TRINITY_DN19872_c0_g1, TRINITY_DN19885_c0_g1, TRINITY_DN19917_c0_g2, TRINITY_DN19986_c0_g1, TRINITY_DN1999_c0_g2, TRINITY_DN20099_c1_g1, TRINITY_DN200_c0_g1, TRINITY_DN2013_c2_g1, TRINITY_DN2015_c3_g1, TRINITY_DN20219_c0_g1, TRINITY_DN20233_c0_g1, TRINITY_DN2063_c0_g1, TRINITY_DN20644_c1_g1, TRINITY_DN20751_c0_g1, TRINITY_DN20783_c0_g1, TRINITY_DN20801_c0_g1, TRINITY_DN21088_c0_g2, TRINITY_DN21187_c0_g1, TRINITY_DN21260_c0_g1, TRINITY_DN2127_c0_g1, TRINITY_DN2128_c0_g1, TRINITY_DN21362_c0_g1, TRINITY_DN2145_c0_g1, TRINITY_DN21460_c0_g1, TRINITY_DN21548_c0_g1, TRINITY_DN21573_c0_g1, TRINITY_DN21705_c0_g2, TRINITY_DN21770_c0_g1, TRINITY_DN21789_c0_g1, TRINITY_DN21794_c0_g1, TRINITY_DN22098_c0_g1, TRINITY_DN39693_c0_g1, TRINITY_DN4125_c0_g2, TRINITY_DN22153_c0_g1, TRINITY_DN22303_c0_g1, TRINITY_DN22632_c0_g1, TRINITY_DN22701_c0_g1, TRINITY_DN22730_c0_g1, TRINITY_DN4921_c0_g1, TRINITY_DN227_c1_g1, TRINITY_DN227_c1_g2, TRINITY_DN22827_c0_g1, TRINITY_DN23144_c0_g1, TRINITY_DN2333_c0_g1, TRINITY_DN23561_c0_g1, TRINITY_DN23582_c0_g2, TRINITY_DN23787_c0_g2, TRINITY_DN2381_c0_g1, TRINITY_DN24074_c0_g1, TRINITY_DN24220_c0_g1, TRINITY_DN2425_c2_g1, TRINITY_DN2437_c1_g1, TRINITY_DN24550_c0_g1, TRINITY_DN2460_c1_g1, TRINITY_DN247_c1_g1, TRINITY_DN25403_c0_g1, TRINITY_DN25413_c0_g1, TRINITY_DN25433_c0_g1, TRINITY_DN25443_c0_g2, TRINITY_DN25651_c0_g2, TRINITY_DN25664_c0_g1, TRINITY_DN2570_c0_g1, TRINITY_DN25919_c0_g1, TRINITY_DN259_c2_g1, TRINITY_DN26066_c0_g1, TRINITY_DN26301_c0_g2, TRINITY_DN2643_c0_g2, TRINITY_DN26816_c0_g2, TRINITY_DN27241_c0_g1, TRINITY_DN27255_c0_g1, TRINITY_DN2746_c0_g1, TRINITY_DN2747_c0_g1, TRINITY_DN2752_c1_g1, TRINITY_DN6234_c0_g1, TRINITY_DN2801_c0_g2, TRINITY_DN2810_c0_g1, TRINITY_DN28156_c0_g1, TRINITY_DN2821_c1_g1, TRINITY_DN2838_c2_g1, TRINITY_DN28460_c0_g2, TRINITY_DN28536_c0_g1, TRINITY_DN28576_c0_g1, TRINITY_DN28598_c0_g1, TRINITY_DN28699_c0_g1, TRINITY_DN28718_c0_g1, TRINITY_DN2885_c0_g1, TRINITY_DN2909_c0_g2, TRINITY_DN29193_c0_g1, TRINITY_DN294_c0_g1, TRINITY_DN2_c6_g1, TRINITY_DN3033_c1_g2, TRINITY_DN30538_c0_g1,	ATP synthase subunit C lysine N-methyltransferase [*H. sapiens*], Olfactory receptor class A-like protein 1 [*D. rerio*], Atrial natriuretic peptide receptor 2 [*A. japonica*], Sphingosine-1-phosphate phosphatase 2 [*H. sapiens*], Interleukin-31 receptor subunit alpha [*H. sapiens*], Cytochrome c oxidase subunit 6C-1 [*T. obesus*], Thymic stromal cotransporter homolog [*C. familiaris*], Piezo-type mechanosensitive ion channel component 2 [*M. musculus*], Mitochondrial inner membrane protease subunit 2 [*D. rerio*], XK-related protein 5 [*H. sapiens*], Immunoglobulin superfamily member 11 [*B. taurus*], Small integral membrane protein 4 [*H. sapiens*], Polypeptide N-acetylgalactosaminyltransferase 17 [*H. sapiens*], Transmembrane protein 245 [*H. sapiens*], Cadherin EGF LAG seven-pass G-type receptor 1 [*M. musculus*], Endothelin receptor type B [*S. scrofa*], Retrotransposon-like protein 1 [*B. taurus*], Endosome/lysosome-associated apoptosis and autophagy regulator family member 2 [*X. laevis*], Mitochondrial uncoupling protein 2 [*P. abelii*], Claudin-7-A [*D. rerio*], Lipase maturation factor 1 [*B. taurus*], Nuclear envelope integral membrane protein 2 [*H. sapiens*], Kin of IRRE-like protein 3 [*H. sapiens*], Solute carrier family 35 member C2 [*M. musculus*], Cytochrome c oxidase subunit NDUFA4 [*D. rerio*], Reticulon-2 [*M. musculus*], Carbonic anhydrase 9 [*M. musculus*], Heparan sulfate glucosamine 3-O-sulfotransferase 5 [*H. sapiens*], Excitatory amino acid transporter 2 [*R. norvegicus*], Very-long-chain 3-oxoacyl-CoA reductase [*A. boschas*], Epoxide hydrolase 4 [*M. musculus*], Alpha-N-acetylneuraminide alpha-2,8-sialyltransferase [*B. taurus*], Transmembrane protein 237A [*D. rerio*], Tetraspanin-11 [*B. taurus*], Transmembrane protein 130 [*H. sapiens*], Deleted in malignant brain tumors 1 protein [*R. norvegicus*], Protein O-mannosyl-transferase 1 [*D. rerio*], Solute carrier family 45 member 4 [*H. sapiens*], Putative membrane protein Bcell_0381 [*B. cellulosilyticus*], Polypeptide N-acetylgalactosaminyltransferase 3 [*H. sapiens*], Desmocollin-2 [*M. musculus*], Acid-sensing ion channel 2 [*D. rerio*], Sodium/glucose cotransporter 1 [*R. norvegicus*], NADH dehydrogenase 1 beta subcomplex subunit 6 [*P. pygmaeus*]<br>Multidrug and toxin extrusion protein 1 [*D. rerio*], Zinc transporter 2 [*M. musculus*], Transcription termination factor 4 [*R. norvegicus*], WD repeat-containing protein 70 [*X. tropicalis*], Polycystic kidney disease protein 1-like 2 [*H. sapiens*], Protein RIC-3 [*X. tropicalis*], Netrin receptor UNC5D [*H. sapiens*], Protein TIC 214 [*P. koraiensis*], Proteinase-activated receptor 1 [*B. taurus*], DNAJC30 [*H. sapiens*], Neurensin-1 [*M. musculus*], Glycerophosphodiester phosphodiesterase 1 [*B. taurus*], Prostaglandin F2 receptor negative regulator [*H. sapiens*], Ryanodine receptor 3 [*M. musculus*], Protein O-mannose kinase [*D. rerio*], Calcium-binding protein 8 [*H. sapiens*], CD276 antigen [*R. norvegicus*], ER membrane protein complex subunit 4 [*D. rerio*], Corticotropin-releasing factor receptor 2 [*X. laevis*], Tumor necrosis factor ligand superfamily member 15 [*R. norvegicus*], Type-1 angiotensin II receptor-associated protein [*P. abelii*], Selenoprotein K [*D. rerio*],

	TRINITY_DN3077_c0_g1, TRINITY_DN3093_c0_g2, TRINITY_DN30995_c0_g1, TRINITY_DN310_c1_g1, TRINITY_DN3112_c0_g1, TRINITY_DN3149_c0_g1, TRINITY_DN31802_c0_g2, TRINITY_DN3218_c2_g1, TRINITY_DN3219_c0_g1, TRINITY_DN3220_c0_g1, TRINITY_DN3257_c1_g1, TRINITY_DN3260_c0_g1, TRINITY_DN32655_c1_g1, TRINITY_DN3303_c0_g1, TRINITY_DN3303_c1_g1, TRINITY_DN33397_c0_g1, TRINITY_DN33551_c1_g1, TRINITY_DN3386_c0_g1, TRINITY_DN3438_c0_g1, TRINITY_DN34573_c0_g1, TRINITY_DN5121_c0_g2, TRINITY_DN3462_c0_g1, TRINITY_DN34764_c0_g1, TRINITY_DN3527_c0_g1, TRINITY_DN3573_c0_g1, TRINITY_DN36018_c0_g1, TRINITY_DN36235_c0_g1, TRINITY_DN3660_c1_g1, TRINITY_DN3689_c0_g1, TRINITY_DN3707_c0_g1, TRINITY_DN37195_c0_g1, TRINITY_DN37219_c0_g1, TRINITY_DN3733_c0_g1, TRINITY_DN3934_c0_g1, TRINITY_DN3941_c0_g1, TRINITY_DN3948_c0_g1, TRINITY_DN3963_c0_g1, TRINITY_DN40073_c0_g1, TRINITY_DN4017_c0_g1, TRINITY_DN4047_c0_g1, TRINITY_DN40557_c0_g2, TRINITY_DN41705_c0_g1, TRINITY_DN419_c0_g2, TRINITY_DN42321_c0_g2, TRINITY_DN4248_c0_g1, TRINITY_DN42524_c0_g1, TRINITY_DN4261_c1_g1, TRINITY_DN43746_c0_g1, TRINITY_DN4399_c0_g3, TRINITY_DN439_c0_g2, TRINITY_DN4400_c0_g1, TRINITY_DN4411_c0_g1, TRINITY_DN4423_c0_g1, TRINITY_DN4456_c1_g1, TRINITY_DN45456_c0_g1, TRINITY_DN45456_c0_g2, TRINITY_DN45653_c1_g1, TRINITY_DN460_c0_g1, TRINITY_DN4622_c1_g1, TRINITY_DN4627_c0_g1, TRINITY_DN46806_c0_g1, TRINITY_DN47232_c0_g3, TRINITY_DN47374_c0_g1, TRINITY_DN4751_c3_g2, TRINITY_DN4777_c0_g1, TRINITY_DN4952_c0_g1, TRINITY_DN4957_c0_g1, TRINITY_DN495_c0_g1, TRINITY_DN496_c0_g2, TRINITY_DN49920_c0_g1, TRINITY_DN4994_c0_g1, TRINITY_DN5011_c0_g1, TRINITY_DN5252_c0_g1, TRINITY_DN5043_c0_g1, TRINITY_DN5050_c0_g1, TRINITY_DN5058_c0_g1, TRINITY_DN51083_c0_g1, TRINITY_DN5163_c0_g1, TRINITY_DN5170_c0_g1, TRINITY_DN52309_c0_g1, TRINITY_DN5253_c0_g1, TRINITY_DN53171_c0_g2, TRINITY_DN5320_c0_g1, TRINITY_DN53396_c0_g1, TRINITY_DN535_c1_g2, TRINITY_DN5392_c0_g1, TRINITY_DN5398_c0_g1, TRINITY_DN545_c0_g1, TRINITY_DN5480_c0_g1, TRINITY_DN5498_c0_g1, TRINITY_DN55099_c0_g1, TRINITY_DN55384_c0_g2, TRINITY_DN55450_c0_g1, TRINITY_DN5588_c0_g1, TRINITY_DN5630_c0_g1, TRINITY_DN5648_c0_g1, TRINITY_DN5706_c3_g1, TRINITY_DN5763_c0_g1, TRINITY_DN5853_c0_g1, TRINITY_DN587_c0_g1, TRINITY_DN58956_c0_g2, TRINITY_DN6010_c1_g1, TRINITY_DN602_c0_g1, TRINITY_DN6058_c0_g1, TRINITY_DN6118_c0_g1, TRINITY_DN6120_c0_g1, TRINITY_DN612_c0_g1, TRINITY_DN6269_c0_g1, TRINITY_DN6271_c0_g1, TRINITY_DN6288_c0_g1, TRINITY_DN6337_c0_g1, TRINITY_DN6397_c1_g1, TRINITY_DN6411_c0_g1, TRINITY_DN6417_c0_g1, TRINITY_DN6424_c0_g1, TRINITY_DN6476_c1_g1, TRINITY_DN6492_c0_g1, TRINITY_DN6517_c0_g1, TRINITY_DN6569_c0_g1, TRINITY_DN66508_c0_g1, TRINITY_DN6659_c0_g1, TRINITY_DN6683_c0_g1, TRINITY_DN674_c1_g1,	Histamine H3 receptor [*M. musculus*], Retinol dehydrogenase 12 [*H. sapiens*], Palmitoyltransferase ZDHHC4 [*D. rerio*], Cytochrome c oxidase subunit 7B [*H. sapiens*], Cytochrome c oxidase subunit 8A [*C. syrichta*], Leucine-rich repeat and immunoglobulin-like domain-containing nogo receptor-interacting protein 1 [*G. gallus*], Presenilins-associated rhomboid-like protein [*B. taurus*], Arachidonate 5-lipoxygenase-activating protein [M. fascicularis], E3 ubiquitin-protein ligase RNF19A [*S. scrofa*], Transient receptor potential cation channel subfamily V member 1 [*R. norvegicus*], Probable G-protein coupled receptor 156 [*H. sapiens*], RPE-retinal G protein-coupled receptor [*B. taurus*], Carbohydrate sulfotransferase 1 [*D. rerio*], NADH dehydrogenase 1 beta subcomplex subunit 8 [*B. taurus*], Dolichyl-phosphate beta-glucosyltransferase [*M. musculus*], Olfactory receptor 52B2 [*H. sapiens*], Solute carrier family 12 member 2 [*S. acanthias*], Syntaxin-2 [*H. sapiens*], Sodium/calcium exchanger 1 [*M. musculus*], Plexin-A2 [*H. sapiens*], Polypeptide N-acetylgalactosaminyltransferase-like 6 [*H. sapiens*], DNA damage-regulated autophagy modulator protein 1 [*H. sapiens*], Opsin-5 [*M. musculus*], Inward rectifier potassium channel 16 [*R. norvegicus*], Trophoblast glycoprotein [*M. musculus*], Small integral membrane protein 20 [*H. sapiens*], Lecithin retinol acyltransferase [*B. taurus*], Kappa-type opioid receptor [*C. porcellus*], Flavin-containing monooxygenase 5 [*R. norvegicus*], Syntaxin-8 [*H. sapiens*], Protein MAL2 [*B. taurus*], Transmembrane and coiled-coil domain-containing protein 6 [*M. musculus*], Transmembrane protein 258 [*X. tropicalis*], Solute carrier family 23 member 1 [*H. sapiens*], Receptor-type tyrosine-protein phosphatase N2 [*M. musculus*], LRP11 [*H. sapiens*],3-b-hydroxysteroid-D(8),D(7)-isomerase [*H. sapiens*], Endoplasmic reticulum aminopeptidase 2 [*B. taurus*], Probable C-mannosyltransferase DPY19L3 [*H. sapiens*], E3 ubiquitin-protein ligase RNF130 [*R. norvegicus*], Receptor activity-modifying protein 1 [*C. porcellus*], Retinol dehydrogenase 11 [*H. sapiens*], Solute carrier family 35 member E3 [*D. rerio*], NADH dehydrogenase 1 beta subcomplex subunit 1 [*P. troglodytes*], Protein C3orf33 [*H. sapiens*], Polypeptide N-acetylgalactosaminyltransferase 9 [*H. sapiens*], Transmembrane protein 53-B [*X. laevis*], Potassium channel subfamily T member 2 [*H. sapiens*], NADH dehydrogenase 1 beta subcomplex subunit 3 [*P. pygmaeus*], Putative protein 2 [*T. rubripes*], Gamma-secretase subunit APH-1A [*M. musculus*], Contactin-associated protein 1 [*H. sapiens*], Thyrotropin receptor [*I. punctatus*], Protein transport protein Sec61 subunit gamma [*G. morhua*], SIA4C [*R. norvegicus*], RH2a [P. falciparum], TACD2 [*M. musculus*], P2Y purinoceptor 8 [*G. gallus*], CSC1-like protein 1 [*P. abelii*], Opsin-3 [*M. musculus*], LFN1L [*D. rerio*], COX8B [*E. fulvus*], Transmembrane protein 108 [*H. sapiens*], Transmembrane protein 238 [*M. musculus*], Aquaporin-12B [*H. sapiens*], Seizure protein 6 homolog [*X. laevis*], ARMD3 [*D. rerio*], APMAP [*H. sapiens*], Claudin-3 [*R. norvegicus*], EPCAM [*G. gallus*], Adenylate cyclase type 7 [*H. sapiens*], MSPD1 [*B. taurus*], KCNG1 [*H. sapiens*], ADCK5 [*M.musculus*], Aminopeptidase N [*R. norvegicus*], TOM5 [*M.*

	TRINITY_DN67624_c0_g1, TRINITY_DN6767_c0_g1, TRINITY_DN6767_c0_g2, TRINITY_DN68016_c0_g3, TRINITY_DN6808_c0_g1, TRINITY_DN6871_c1_g1, TRINITY_DN689_c0_g2, TRINITY_DN6910_c0_g1, TRINITY_DN694_c0_g2, TRINITY_DN6969_c0_g2, TRINITY_DN70008_c0_g1, TRINITY_DN7070_c0_g1, TRINITY_DN7086_c0_g1, TRINITY_DN7119_c2_g1, TRINITY_DN7125_c0_g1, TRINITY_DN7149_c0_g2, TRINITY_DN7202_c0_g1, TRINITY_DN7237_c0_g1, TRINITY_DN7238_c0_g2, TRINITY_DN7250_c0_g1, TRINITY_DN7250_c0_g2, TRINITY_DN7253_c0_g1, TRINITY_DN7273_c0_g2, TRINITY_DN7303_c0_g1, TRINITY_DN7305_c1_g1, TRINITY_DN7305_c1_g2, TRINITY_DN7323_c0_g1, TRINITY_DN7378_c0_g1, TRINITY_DN7409_c0_g1, TRINITY_DN7442_c0_g1, TRINITY_DN7442_c0_g2, TRINITY_DN748_c0_g2, TRINITY_DN7512_c0_g1, TRINITY_DN7529_c0_g1, TRINITY_DN7582_c0_g1, TRINITY_DN7685_c0_g2, TRINITY_DN769_c0_g1, TRINITY_DN7708_c0_g1, TRINITY_DN7709_c0_g1, TRINITY_DN770_c0_g2, TRINITY_DN7740_c0_g2, TRINITY_DN7769_c0_g1, TRINITY_DN8016_c0_g1, TRINITY_DN7778_c0_g1, TRINITY_DN7787_c1_g2, TRINITY_DN7863_c0_g2, TRINITY_DN7995_c0_g1, TRINITY_DN8012_c0_g1, TRINITY_DN8035_c0_g1, TRINITY_DN8039_c0_g1, TRINITY_DN8057_c0_g2, TRINITY_DN8091_c0_g1, TRINITY_DN8129_c0_g1, TRINITY_DN82313_c0_g1, TRINITY_DN828_c2_g1, TRINITY_DN83218_c0_g1, TRINITY_DN8369_c0_g1, TRINITY_DN843_c2_g1, TRINITY_DN8446_c0_g1, TRINITY_DN8460_c0_g1, TRINITY_DN84_c0_g1, TRINITY_DN8502_c0_g1, TRINITY_DN8592_c0_g1, TRINITY_DN8644_c0_g1, TRINITY_DN8665_c0_g1, TRINITY_DN8684_c0_g1, TRINITY_DN8728_c0_g1, TRINITY_DN8766_c0_g1, TRINITY_DN8786_c0_g1, TRINITY_DN8786_c0_g2, TRINITY_DN8839_c0_g1, TRINITY_DN8847_c0_g1, TRINITY_DN8866_c0_g1, TRINITY_DN8869_c0_g1, TRINITY_DN8877_c0_g1, TRINITY_DN8915_c0_g1, TRINITY_DN8959_c0_g1, TRINITY_DN89762_c0_g1, TRINITY_DN9013_c0_g1, TRINITY_DN9056_c0_g1, TRINITY_DN9067_c0_g1, TRINITY_DN906_c0_g1, TRINITY_DN909_c0_g1, TRINITY_DN9112_c0_g1, TRINITY_DN9130_c0_g1, TRINITY_DN9170_c0_g1, TRINITY_DN9413_c0_g2, TRINITY_DN9452_c0_g1, TRINITY_DN9482_c0_g1, TRINITY_DN9514_c0_g1, TRINITY_DN9515_c0_g1, TRINITY_DN9518_c0_g2, TRINITY_DN9585_c0_g1, TRINITY_DN9632_c1_g1, TRINITY_DN9632_c1_g2, TRINITY_DN978_c0_g1, TRINITY_DN984_c0_g1, TRINITY_DN992_c0_g1, TRINITY_DN9941_c0_g2, TRINITY_DN995_c0_g1, TRINITY_DN9971_c0_g1, TRINITY_DN9982_c0_g2	*musculus*], VAPB [*B. taurus*], Transmembrane protein 141 [*B. taurus*], SIM29 [*H. sapiens*], OXA1L [*M. musculus*], Netrin receptor UNC5A [R. norvegcus], GGT5 [*M. musculus*], PARL [*B. taurus*], CXCR4 [*B. taurus*], PIGF [*M. musculus*], Kinocilin [*M. musculus*], Solute carrier family 35 member F6 [*H. sapiens*], S6A20 [*H. sapiens*], Glycophorin-C [*H. sapiens*], DAD1 [*R. norvegicus*], AT1A3 [*G. gallus*], PAQRB [*R. norvegicus*], Solute carrier family 25 member 33 [*D. rerio*], Cell adhesion molecule 4 [*X. laevis*], NADH dehydrogenase 1 alpha subcomplex subunit 11 [*M. musculus*], Metalloreductase STEAP4 [*H. sapiens*], CDKAL [*X. laevis*], TGBR3 [*S. scrofa*], VTM2A [*M. musculus*], Cytochrome P450 2F3 [*C. hircus*], Tim23 [*D. rerio*], Protein odr-4 homolog [*M. musculus*], Aquaporin-1 [*P. abelii*], GRM3 [*R. norvegicus*], Gper1 [*D. rerio*], LRRT4 [*M. musculus*], SFT2C [*M. musculus*], CSC1-like protein 2 [*M. musculus*], Tricarboxylate transport protein B [*D. rerio*], B3GALT5 [*G. gorilla*], CISD1 [*M. musculus*], Uroplakin-3b [*M. musculus*], GPAT2 [*M. musculus*], ATPK [*M. musculus*], Transmembrane protein 256 [*D. rerio*], Complex I assembly factor TIMMDC1 [*D. rerio*], Integrin alpha-L [*H. sapiens*], TAS1R3 [*G. gorilla*], LYVE1 [*M. musculus*], ACKMT [*X. laevis*], CD40 [*C. jacchus*], CKLF4 [*M. musculus*], Sodium/potassium-transporting ATPase subunit alpha-2 [*G. gallus*], PLCA [*H. sapiens*], Interleukin-22 receptor subunit alpha-2 [*R. norvegicus*], Transmembrane protein 131-like [*H. sapiens*], UQCRFS1 [*S. syndactylus*], Ceramide kinase [*H. sapiens*], Fibrocystin-L [*H. sapiens*], Sodium/hydrogen exchanger 9B2 [*H. sapiens*], ASML [*H. sapiens*], Sialomucin core protein 24 [*R. norvegicus*], PLS1 [*H. sapiens*], S5A1 [*M. musculus*], NADH dehydrogenase 1 alpha subcomplex subunit 13, PTPRM [*M. musculus*], PTPRO [*H. sapiens*], Na(+)/H(+) exchanger beta [*O. mykiss*], OST4 [*R. norvegicus*], GALT6 [*M. musculus*], Zonadhesin [*S. scrofa*], Tetraspanin-14 [*M. musculus*], Steryl-sulfatase [*H. sapiens*], ROMO1 [*X. laevis*], TMTC4 [*M. musculus*], KIAA0319 [*H. sapiens*], Zinc transporter ZIP1 [*B. taurus*], TIM22 [*R. norvegicus*], Marchf5 [*D. rerio*], ALG14 homolog [*R. norvegicus*], Choline transporter-like protein 5-A [*D. rerio*], G-protein coupled receptor 157 [*H. sapiens*], Fukutin-related protein [*H. sapiens*], Small integral membrane protein 11A [*M. musculus*], EVC [*M. musculus*], Transmembrane protein 126A [*M. musculus*], G-protein coupled receptor 26 [*M. musculus*], NOC4L [*D. rerio*], Olfactory receptor 10G4 [*H. sapiens*], Calnexin [*P. abelii*], CD81 antigen [*P. troglodytes*], CD166 antigen homolog A [*D. rerio*], Magnesium transporter NIPA2 [*P. abelii*], AGRL2 [*H. sapiens*], AGRL3 [*H. sapiens*], Olfactory receptor 1030 [*M. musculus*], NADH dehydrogenase 1 beta subcomplex subunit 5 [*B. taurus*], MTCH2 [*P. abelii*], Syntaxin-7 [*R. norvegicus*], Tricarboxylate transport protein A [*D. rerio*], KCNH3 [*R. norvegicus*], CGT [*R. norvegicus*], Delta-like protein 4 [*H. sapiens*], C18orf19 homolog A [*D. rerio*], COX8B [*C. syrichta*], SYN1L [*D. rerio*], T-cell antigen CD7 [*M. musculus*], Protein huluwa [*D. rerio*], RNFT1 [*D. rerio*], ANPRA [*R. norvegicus*], TOM7 homolog [*S. scrofa*], GHITM [*H. sapiens*], Transmembrane protein 212 [*H. sapiens*], MMGT1 [*D. rerio*], G6PT1 [*H. sapiens*], TRPM3 [*H. sapiens*], Protein Mpv17

		[*D. rerio*], CCGL [*M. musculus*], Sugar transporter SWEET1 [*D. rerio*], Protein ccsmst1 [*X. tropicalis*], ASPH [*B. taurus*], PLAT3 [*H. sapiens*], HIG1 domain family member 2A [*M. musculus*], NADH dehydrogenase 1 subunit C1 [*B. taurus*], DNJC1 [*M. musculus*], Protein jagged-2 [*H. sapiens*], Protein FAM162B [*D. rerio*], GPAM [*S. scrofa*], NSDHL [*H. sapiens*], Transmembrane protein 41A-A [*D. rerio*], TGO1 [*M. musculus*], Semaphorin-6D [*H. sapiens*], MGAT2 [*H. sapiens*], NADH dehydrogenase 1 alpha subcomplex subunit 1 [*M. musculus*], MALD3 [*H. sapiens*], MFS10 [*B. taurus*], LRC3B [*D. rerio*], Transmembrane protein 223 [*D. rerio*], EMC10 [*D. rerio*], TX264 [*H. sapiens*], GJB1 [*E. caballus*], HS2ST [*G. gallus*], U2AFM [*H. sapiens*], ATPMD [*H. sapiens*], Soluble calcium-activated nucleotidase 1 [*R. norvegicus*], Tafazzin [*M. mulatta*], NADH dehydrogenase 1 alpha subcomplex subunit 3 [*M. musculus*], Polyprenol reductase [*X. tropicalis*], QCR10 [*B. taurus*], GPAA1 [*M. musculus*], LPCAT4 [*X. tropicalis*], Prefoldin subunit 1 [*D. rerio*], RNFT2 [*M. musculus*], COX14 homolog [*D. rerio*], Solute carrier family 12 member 8 [*X. laevis*], Metaxin-1 [*S. scrofa*], Monocarboxylate transporter 6 [*M. musculus*]
MF identical protein binding, GO:0042802, *q* value <0.0001	TRINITY_DN10195_c0_g1, TRINITY_DN10309_c0_g1, TRINITY_DN1064_c1_g1, TRINITY_DN2857_c0_g1, TRINITY_DN10726_c0_g1, TRINITY_DN10903_c0_g1, TRINITY_DN10903_c0_g2, TRINITY_DN10962_c0_g1, TRINITY_DN11172_c0_g1, TRINITY_DN11487_c0_g1, TRINITY_DN11517_c0_g1, TRINITY_DN1151_c1_g1, TRINITY_DN1170_c0_g3, TRINITY_DN12007_c0_g1, TRINITY_DN12015_c0_g1, TRINITY_DN12050_c0_g1, TRINITY_DN12182_c0_g1, TRINITY_DN12269_c0_g1, TRINITY_DN12284_c0_g1, TRINITY_DN12375_c0_g1, TRINITY_DN12417_c0_g1, TRINITY_DN13430_c2_g1, TRINITY_DN27787_c3_g1, TRINITY_DN30191_c0_g1, TRINITY_DN30893_c0_g1, TRINITY_DN33430_c0_g1, TRINITY_DN42994_c0_g1, TRINITY_DN42527_c0_g1, TRINITY_DN43152_c0_g1, TRINITY_DN43034_c0_g1, TRINITY_DN44389_c2_g1, TRINITY_DN45528_c1_g1, TRINITY_DN45909_c0_g1, TRINITY_DN1260_c0_g2, TRINITY_DN1264_c0_g1, TRINITY_DN1272_c1_g1, TRINITY_DN1272_c1_g2, TRINITY_DN1275_c2_g1, TRINITY_DN13133_c0_g1, TRINITY_DN13161_c0_g1, TRINITY_DN13289_c0_g1, TRINITY_DN132_c0_g1, TRINITY_DN13517_c0_g1, TRINITY_DN14226_c1_g1, TRINITY_DN14367_c0_g1, TRINITY_DN1524_c0_g1, TRINITY_DN15535_c0_g1, TRINITY_DN16019_c0_g1, TRINITY_DN16144_c0_g1, TRINITY_DN162_c0_g2, TRINITY_DN16324_c0_g1, TRINITY_DN1638_c0_g1, TRINITY_DN16563_c0_g1, TRINITY_DN16576_c1_g1, TRINITY_DN1707_c0_g1, TRINITY_DN17631_c0_g1, TRINITY_DN17699_c0_g1, TRINITY_DN1773_c1_g1, TRINITY_DN17780_c0_g1, TRINITY_DN1830_c1_g1, TRINITY_DN1877_c0_g1, TRINITY_DN19422_c0_g1, TRINITY_DN19429_c0_g1, TRINITY_DN1958_c2_g2, TRINITY_DN20070_c0_g1, TRINITY_DN20155_c0_g1, TRINITY_DN20705_c2_g1, TRINITY_DN20751_c0_g3, TRINITY_DN207_c1_g1, TRINITY_DN2095_c0_g1, TRINITY_DN2105_c0_g1, TRINITY_DN2105_c0_g2, TRINITY_DN2128_c0_g1, TRINITY_DN2130_c2_g1, TRINITY_DN2130_c2_g2,	Septin-2 [*R. norvegicus*], NCLX [*M. musculus*], DNA repair protein XRCC4 [*H. sapiens*], Membrane protein MLC1 [*M. musculus*], ATP-dependent DNA helicase Q5 [*R. norvegicus*], Claudin-like protein ZF-A89 [*D. rerio*], SMAD1 [*R. norvegicus*], Receptor-type tyrosine-protein phosphatase mu [*H. sapiens*], Glutamate receptor 4 [*R. norvegicus*], RNA-binding protein Musashi homolog 2 [*H. sapiens*], Homeobox-containing protein 1 [*H. sapiens*], Ryanodine receptor 2 [*R. norvegicus*], Fibrinogen gamma chain [*H. sapiens*], HS90A [*G. gallus*], Nucleoside diphosphate kinase 6 [*D. rerio*], Zona pellucida sperm-binding protein 3 [*G. gallus*], Thymidine kinase, cytosolic [*G. gallus*], Collagen alpha-1(VII) chain [*H. sapiens*], Annexin A4 [*B. taurus*], E3 ubiquitin-protein ligase RNF31 [*H. sapiens*], Claudin-14 [*H. sapiens*], KCNH2 [*R. norvegicus*], Toll/interleukin-1 receptor domain-containing adapter protein [*H. sapiens*], Ubiquitin carboxyl-terminal hydrolase 2 [*H. sapiens*], Nicotinamide phosphoribosyltransferase [*S. scrofa*], GDP-L-fucose synthase [*H. sapiens*], P2X purinoceptor 4 [*H. sapiens*], CCAAT/enhancer-binding protein gamma [*H. sapiens*], Interleukin-31 receptor subunit alpha [*H. sapiens*], PUF60 [*R. norvegicus*], Cyclic AMP-dependent transcription factor ATF-3 [*M. musculus*], Platelet-derived growth factor subunit B [*M. musculus*], KHDR1 [*R. norvegicus*], DNA replication licensing factor MCM6 [*R. norvegicus*], Protein kinase C-binding protein NELL2 [*R. norvegicus*], Ryanodine receptor 3 [*M. musculus*], POLR1B [*P. abelii*], E3 ubiquitin-protein ligase CCNB1IP1 [*H. sapiens*], Dynein light chain 2 [*R. norvegicus*], Zona pellucida sperm-binding protein 4 [*R. norvegicus*], Spliceosome RNA helicase DDX39B [*B. taurus*], Exosome complex component RRP43 [*M. musculus*], Protein PAXX [*H. sapiens*], RAB31 [*H. sapiens*], Ornithine aminotransferase [*B. taurus*], Fructose-1,6-bisphosphatase isozyme 2 [*R. norvegicus*], THAP domain-containing protein 7 [*H. sapiens*], Uridine

	TRINITY_DN2131_c0_g1, TRINITY_DN21830_c0_g1, TRINITY_DN22081_c0_g1, TRINITY_DN2209_c0_g1, TRINITY_DN22303_c0_g1, TRINITY_DN22730_c0_g1, TRINITY_DN2280_c0_g1, TRINITY_DN2297_c1_g1, TRINITY_DN23143_c0_g1, TRINITY_DN23182_c0_g1, TRINITY_DN23223_c0_g2, TRINITY_DN2335_c0_g1, TRINITY_DN2345_c3_g1, TRINITY_DN2351_c0_g1, TRINITY_DN23965_c0_g1, TRINITY_DN2403_c0_g2, TRINITY_DN24550_c0_g1, TRINITY_DN28673_c0_g1, TRINITY_DN30537_c0_g1, TRINITY_DN25123_c3_g1, TRINITY_DN25403_c0_g1, TRINITY_DN25608_c0_g2, TRINITY_DN2573_c0_g2, TRINITY_DN26031_c0_g2, TRINITY_DN26301_c0_g2, TRINITY_DN2673_c0_g1, TRINITY_DN2789_c0_g2, TRINITY_DN2797_c1_g1, TRINITY_DN2817_c0_g2, TRINITY_DN2849_c0_g1, TRINITY_DN29294_c0_g1, TRINITY_DN2988_c0_g1, TRINITY_DN3081_c0_g1, TRINITY_DN3139_c1_g1, TRINITY_DN3254_c0_g1, TRINITY_DN3254_c1_g1, TRINITY_DN32726_c0_g1, TRINITY_DN3324_c0_g1, TRINITY_DN3324_c0_g2, TRINITY_DN33503_c0_g1, TRINITY_DN3392_c0_g2, TRINITY_DN34145_c0_g1, TRINITY_DN3491_c0_g1, TRINITY_DN35293_c0_g1, TRINITY_DN35952_c0_g2, TRINITY_DN36018_c0_g1, TRINITY_DN36465_c0_g1, TRINITY_DN366_c0_g1, TRINITY_DN36964_c0_g1, TRINITY_DN36998_c0_g1, TRINITY_DN37115_c0_g1, TRINITY_DN37324_c0_g1, TRINITY_DN37422_c0_g1, TRINITY_DN37519_c0_g1, TRINITY_DN37884_c0_g1, TRINITY_DN3844_c0_g1, TRINITY_DN39077_c1_g1, TRINITY_DN39130_c0_g1, TRINITY_DN39132_c0_g1, TRINITY_DN39201_c0_g1, TRINITY_DN3957_c0_g1, TRINITY_DN40157_c0_g2, TRINITY_DN4018_c0_g2, TRINITY_DN403_c1_g1, TRINITY_DN40557_c0_g2, TRINITY_DN41257_c0_g1, TRINITY_DN4128_c0_g1, TRINITY_DN41413_c0_g1, TRINITY_DN4184_c1_g1, TRINITY_DN4206_c0_g2, TRINITY_DN42814_c4_g1, TRINITY_DN4283_c0_g3, TRINITY_DN42986_c0_g1, TRINITY_DN43275_c0_g1, TRINITY_DN44066_c0_g1, TRINITY_DN4462_c0_g1, TRINITY_DN4483_c1_g1, TRINITY_DN44906_c0_g1, TRINITY_DN4527_c0_g1, TRINITY_DN45383_c2_g1, TRINITY_DN4547_c0_g1, TRINITY_DN45653_c1_g1, TRINITY_DN45750_c0_g1, TRINITY_DN4606_c0_g1, TRINITY_DN46461_c0_g1, TRINITY_DN46806_c0_g1, TRINITY_DN468_c0_g2, TRINITY_DN4713_c0_g1, TRINITY_DN47675_c0_g1, TRINITY_DN48090_c1_g1, TRINITY_DN48103_c0_g1, TRINITY_DN4819_c1_g1, TRINITY_DN4872_c0_g1, TRINITY_DN4889_c0_g2, TRINITY_DN4921_c0_g1, TRINITY_DN493_c0_g3, TRINITY_DN496_c0_g2, TRINITY_DN4_c2_g1, TRINITY_DN50310_c0_g1, TRINITY_DN50361_c0_g1, TRINITY_DN5037_c0_g1, TRINITY_DN50559_c0_g1, TRINITY_DN50651_c0_g1, TRINITY_DN50767_c0_g1, TRINITY_DN50769_c0_g1, TRINITY_DN50771_c0_g1, TRINITY_DN50804_c0_g1, TRINITY_DN5082_c1_g1, TRINITY_DN5091_c0_g1, TRINITY_DN51047_c0_g1, TRINITY_DN51167_c0_g1, TRINITY_DN51378_c0_g1, TRINITY_DN51426_c1_g1, TRINITY_DN51570_c1_g1, TRINITY_DN51961_c0_g1, TRINITY_DN521_c0_g1, TRINITY_DN52492_c0_g1, TRINITY_DN5289_c0_g1, TRINITY_DN52908_c0_g1, TRINITY_DN53521_c0_g1, TRINITY_DN53733_c0_g1, TRINITY_DN5412_c0_g1, TRINITY_DN5494_c0_g1,	phosphorylase 2 [*H. sapiens*], Dihydropyrimidinase [*R. norvegicus*], Zinc finger protein 3 [*H. sapiens*], Isocitrate dehydrogenase [NADP] cytoplasmic [*M. ochrogaster*], Purine nucleoside phosphorylase [*R. norvegicus*]Activated RNA polymerase II transcriptional coactivator p15 [*G. gallus*], Protein chibby homolog 1 [*H. sapiens*], Leucine zipper transcription factor-like protein 1 [*R. norvegicus*], Iron-sulfur cluster co-chaperone protein HscB [*M. musculus*], Branched-chain-amino-acid aminotransferase [*H. sapiens*], Claudin-3 [*R. norvegicus*], Interleukin-6 receptor subunit beta [*H. sapiens*], Protein dpy-30 homolog [*H. sapiens*], Cytotoxic and regulatory T-cell molecule [*M. musculus*], Telomere zinc finger-associated protein [*M. musculus*], 40S ribosomal protein S19 [*P. abelii*], Protein regulator of cytokinesis 1 [*H. sapiens*], C-X-C chemokine receptor type 4 [*B. taurus*], GEX1 [*A. thaliana*], Mitochondrial ubiquitin ligase activator of nfkb 1-A [*D. rerio*], KH domain-containing, RNA-binding, signal transduction-associated protein 1 [*G. gallus*], E3 ubiquitin-protein ligase Midline-1 [*M. spretus*], Inhibitor of nuclear factor kappa-B kinase subunit beta [*M. musculus*], Arrestin domain-containing protein 1 [*M. musculus*], Poly(U)-binding-splicing factor PUF60 [*B. taurus*], Baculoviral IAP repeat-containing protein 5.2 [*X. tropicalis*], V-set and transmembrane domain-containing protein 2A [*M. musculus*], Acylamino-acid-releasing enzyme [*M. musculus*], Aquaporin-1 [*P. abelii*], Transformer-2 protein homolog beta [*R. norvegicus*], Growth arrest and DNA damage-inducible protein GADD45 gamma [*M. musculus*], S-adenosylmethionine synthase isoform type-2 [*R. norvegicus*], Ubiquilin-1 [*H. sapiens*], CD276 antigen [*R. norvegicus*], CDGSH iron-sulfur domain-containing protein 1 [*M. musculus*], Pyruvate carboxylase [*B. taurus*], Zona pellucida sperm-binding protein 1 [*H. sapiens*], RING finger protein 17 [*M. fascicularis*], 3-mercaptopyruvate sulfurtransferase [*H. sapiens*], Uridine diphosphate glucose pyrophosphatase NUDT14 [*M. musculus*], Calcium uniporter protein [*M. musculus*], Nuclear transport factor 2 [*R. norvegicus*], Myc box-dependent-interacting protein 1 [*M. musculus*], Superoxide dismutase [Mn] [*M. fascicularis*], Sodium/hydrogen exchanger 9B2 [*H. sapiens*], 60S ribosomal protein L7 [*G. gallus*], Histone-lysine N-methyltransferase SMYD3 [*M. musculus*], ELAV-like protein 2 [*M. musculus*], von Willebrand factor [*H. sapiens*], Aldehyde dehydrogenase [*R. norvegicus*], Voltage-dependent L-type calcium channel subunit beta-2 [*B. taurus*], Transforming growth factor beta-2 proprotein [*M. musculus*], Programmed cell death protein 6 [*H. sapiens*], Glutamine--fructose-6-phosphate aminotransferase [*R. norvegicus*], MIT domain-containing protein 1 [*R. norvegicus*], Peroxynitrite isomerase THAP4 [*M. musculus*], Nucleoside diphosphate kinase A [*M. musculus*], Formin-binding protein 1 [*H. sapiens*], L-threonine 3-dehydrogenase [*B. taurus*], B-cell lymphoma 6 protein [*H. sapiens*], Mucosa-associated lymphoid, tissue lymphoma translocation protein 1 [*M. musculus*], CD166 antigen homolog A [*D. rerio*], Interferon-induced helicase C domain-containing protein 1 [*H. sapiens*], E3 ubiquitin-protein ligase RAD18 [*M. musculus*], Hexokinase-1 [*H. sapiens*], Nuclear transport factor 2 [*R. norvegicus*], Nucleoside diphosphate kinase B [*R. norvegicus*], Matrin-3 [*M. musculus*], Neuroligin-1 [*R. norvegicus*], Serine dehydratase-like [*M. musculus*], Receptor tyrosine-

	TRINITY_DN5498_c0_g1, TRINITY_DN5506_c0_g1, TRINITY_DN55154_c0_g1, TRINITY_DN55191_c0_g1, TRINITY_DN55532_c0_g1, TRINITY_DN55635_c0_g1, TRINITY_DN5571_c0_g1, TRINITY_DN5578_c0_g2, TRINITY_DN56225_c0_g1, TRINITY_DN5648_c0_g1, TRINITY_DN56493_c0_g1, TRINITY_DN57177_c0_g1, TRINITY_DN58673_c2_g1, TRINITY_DN58956_c0_g2, TRINITY_DN5997_c0_g1, TRINITY_DN6002_c0_g1, TRINITY_DN6162_c0_g2, TRINITY_DN61865_c0_g2, TRINITY_DN6315_c0_g1, TRINITY_DN6319_c0_g1, TRINITY_DN6324_c0_g1, TRINITY_DN634_c1_g1, TRINITY_DN6355_c0_g1, TRINITY_DN63735_c0_g1, TRINITY_DN63_c0_g1, TRINITY_DN648_c1_g1, TRINITY_DN65137_c0_g1, TRINITY_DN65173_c1_g1, TRINITY_DN66094_c0_g1, TRINITY_DN6609_c0_g1, TRINITY_DN660_c2_g1, TRINITY_DN6618_c0_g1, TRINITY_DN66410_c1_g1, TRINITY_DN66889_c0_g1, TRINITY_DN6713_c2_g1, TRINITY_DN67146_c0_g1, TRINITY_DN674_c1_g1, TRINITY_DN6823_c0_g1, TRINITY_DN6907_c1_g1, TRINITY_DN7039_c0_g1, TRINITY_DN710_c0_g2, TRINITY_DN7164_c0_g2, TRINITY_DN71852_c0_g1, TRINITY_DN72535_c0_g1, TRINITY_DN746_c0_g2, TRINITY_DN7483_c0_g2, TRINITY_DN749_c1_g2, TRINITY_DN7579_c0_g1, TRINITY_DN7663_c0_g1, TRINITY_DN7777_c0_g1, TRINITY_DN7903_c0_g2, TRINITY_DN79096_c1_g1, TRINITY_DN807_c1_g2, TRINITY_DN8227_c0_g1, TRINITY_DN82462_c0_g1, TRINITY_DN8284_c0_g1, TRINITY_DN828_c2_g1, TRINITY_DN8358_c0_g1, TRINITY_DN8384_c0_g1, TRINITY_DN83898_c0_g2, TRINITY_DN84101_c0_g1, TRINITY_DN8732_c0_g2, TRINITY_DN8900_c0_g1, TRINITY_DN8923_c1_g1, TRINITY_DN89762_c0_g1, TRINITY_DN9165_c1_g1, TRINITY_DN9229_c0_g1, TRINITY_DN9359_c0_g2, TRINITY_DN9415_c0_g1, TRINITY_DN94276_c0_g1, TRINITY_DN947_c0_g1, TRINITY_DN9927_c1_g1, TRINITY_DN992_c0_g1	protein kinase erbB-3 [*H. sapiens*], Deoxycytidylate deaminase [*R. norvegicus*], Maleylacetoacetate isomerase [*M. musculus*], Sjoegren syndrome nuclear autoantigen 1 homolog [*M. musculus*], Double-strand break repair protein MRE11 [*M. musculus*], Macrophage migration inhibitory factor [*S. scrofa*], Heparan sulfate 2-O-sulfotransferase 1 [*G. gallus*], Ribokinase [*H. sapiens*], Transthyretin [*C. porosus*], Potassium voltage-gated channel subfamily H member 2 [*M. musculus*], ATP-dependent Clp protease proteolytic subunit [*M. musculus*], Box C/D snoRNA protein 1 [*M. musculus*], Transcription initiation factor TFIID subunit 10 [*H. sapiens*], L-xylulose reductase [*M. auratus*], Hepatocyte growth factor [*C. familiaris*], Inositol monophosphatase 1 [*B. taurus*]

Table S2. Differentially expressed genes related to receptor recognition, receptor-mediated endocytosis, vesicle trafficking, and Vtg processing during the transition from the PV stage to the EV stage in *A. australis* ovary. Fold change in expression (log_2FC) and *q* values for each gene are shown. A positive log_2FC indicates up-regulation in the PV stage and down-regulation in the EV stage, while a negative log_2FC means up-regulation in the EV stage and down-regulation in the PV stage. Significant DEGs were considered when showing *q* value <0.05 and $-1 \geq log_2FC \geq 1$, representing a 2-FC. Gene names are based on the *A. anguilla* top hit (*A. anguilla* genome, NCBI RefSeq: GCF_013347855.1, Annotation Release 100) from the BLASTp search of deduced protein sequences retrieved from transcriptome. The read counts (mean ± SEM) are shown for each stage (n = 6 per stage).

**Up-regulated genes during PV-EV transition**							
**Gene ID TRINITY_**	**Annotation**	***A. anguilla* BLASTp hit/gene associated**	**Log_2FC**	***q* value**	**PV reads ± SEM**	**EV reads ± SEM**	**Putative function – Reference**
DN11818_c0_g2	CALM/PICALM [*R. norvegicus*]	Calm/Picalm [XP_035263579.1]/si:ch211-200p22.4	-2.32	<0.01	22.7 ± 5.9	154.5 ± 10.2	Adaptor – in mammals [97]
DN2540_c1_g2	CALM/PICALM [*H. sapiens*]	Calm/Picalm-like [XP_035289267.1]/LOC118235714	-1.94	<0.01	39.8 ± 7.6	213.3 ± 10.0	Adaptor – in mammals [97]
DN4120_c0_g1	NUMB [*H. sapiens*]	Numb [XP_035286847.1]/*numb*	-1.07	<0.01	261.6 ± 48.7	740.0 ± 21.3	Adaptor – in mammals [98]
DN3177_c2_g1	APache [*H. sapiens*]	APache [XP_035270050.1]/*btbd8*	-1.41	<0.01	95.8 ± 22.6	359.5 ± 38.1	AP2 interactor – in mammals [99]
DN7661_c0_g1	epsin-2 [*H. sapiens*]	epsin-2 [XP_035254002.1]/*epn2*	-1.30	<0.01	170.6 ± 44.5	545.3 ± 37.8	Adaptor – review on eukaryotes [100]
DN1565_c1_g1	epsin-2 [*H. sapiens*]	epsin-1-like [XP_035286925.1]/LOC118234478	-1.63	<0.01	127.7 ± 22.0	597.7 ± 98.7	Adaptor – review on eukaryotes [100]
DN7762_c1_g1	AAK1 [*R. norvegicus*]	Aak1-like [XP_035234317.1]/LOC118206161	-1.14	<0.01	1012.1 ± 190.9	2611.8 ± 110.6	Endocytic kinase of adaptor proteins – in mammals [101, 102]
DN3798_c0_g1	HIP1R [*H. sapiens*]	Hip1rb [XP_035236094.1]/*hip1rb*	-1.05	<0.01	1367.1 ± 210.4	3281.4 ± 30.5	Adaptor – in mammals [103]
DN9258_c0_g2	endophilin A3 [*G. gallus*]	endophilin A3-like [XP_035252516.1]/LOC118215681	-1.41	<0.01	95.0 ± 10.5	379.2 ± 32.6	Membrane bending – in mammals [104, 105]

DN2294_c0_g1	sorting nexin 18a [*M. musculus*]	sorting nexin 18a [XP_035235114.1]/*snx18a*	-1.54	<0.01	234.5 ± 52.6	734.2 ± 33.8	Trafficking – in mammals [106]
DN259_c1_g1	sorting nexin 17 [*D. rerio*]	sorting nexin 17 [XP_035279736.1]/*snx17*	-1.28	<0.01	144.7 ± 22.5	598.3 ± 54.5	Trafficking – in mammals [107–109]
DN15680_c0_g1	RIN2 [*M. musculus*]	Rin2 [XP_035256209.1]/*rin2*	-1.12	0.02	31.8 ± 2.4	116.3 ± 28.0	Effector for RAB5 – in mammals [110]
DN2563_c8_g1	RABEP2 [*H. sapiens*]	Rabep2 [XP_035254906.1]/*rabep2*	-1.82	<0.01	191.7 ± 48.5	841.3 ± 61.5	Early endosome – in mammals [111]
DN576_c0_g2	RAB1A [*R. norvegicus*]	ras-related ORAB-1 [XP_035262697.1]/LOC118221592	-1.16	<0.01	147.7 ± 24.0	466.3 ± 21.7	Early endosome – in mammals [112]
**Down-regulated genes during PV-EV transition**							
DN721_c0_g2	cathepsin L1 [*C. familiaris*]	cathepsin La [XP_035235752.1]/*ctsla*	1.77	0.02	939.2 ± 175.7	411.7 ± 119.2	Proteolysis – review on oviparity with focus on teleost fish [94], in *F. heteroclitus* [113]
DN1800_c0_g1	cathepsin D [*C. harengus*]	[PREDICTED] nothepsin [XP_035257715.1]/*nots*	1.29	<0.01	255.2 ± 40.1	167.3 ± 38.3	Proteolysis [92, 114]
**Not differentially expressed between stages**							
DN701_c1_g1	VLDLR [*H. sapiens*]	Vldlr [XP_035247347.1]/*vldlr*	-0.44	0.016	77093.9 ± 3277.1	74027.9 ± 2270.2	Receptor – review on teleosts [6, 115]
DN2157_c0_g1	LRP4 [*R. norvegicus*]	proLrp1-like [XP_035291565.1]/LOC118237195	-0.25	0.04	25991.2 ± 681.5	24698.5 ± 492.6	Receptor – review on teleosts [6, 115]
DN2540_c1_g3	CALM/PICALM [*H. sapiens*]	Calm/Picalma [XP_035288603.1]/*picalma*	-0.74	<0.01	481.6 ± 81.0	1037.3 ± 32.9	Adaptor – in mammals [97]

DN2540_c1_g1	CALM/PICALM [*H. sapiens*]	Calm/Picalmb [XP_035240818.1]/*picalmb*	-0.20	0.33	2475.9 ± 234.7	3346.2 ± 285.7	Adaptor – in mammals [97]
DN518_c0_g1	AAK1 [*B. taurus*]	AAK1-like [XP_035247214.1]/LOC118212882	-0.17	0.44	516.7 ± 69.9	799.8 ± 48.2	Endocytic kinase of adaptor proteins – in mammals [101, 102]
DN8136_c0_g1	endophilin A3 [*G. gallus*]	endophilin A3 [XP_035274525.1]/*sh3gl3a*	-0.94	<0.01	48.5 ± 4.4	142.5 ± 12.6	Membrane bending - in mammals [104, 105]
DN447_c0_g1	clathrin heavy chain 1 [*M. musculus*]	clathrin heavy chain 1 [XP_035235459.1]/*cltcl1*	-0.52	<0.01	5868.5 ± 431.8	8444.3 ± 161.1	Coat protein – in mammals [116], in *Oncorhynchus clarki* [117]
DN69174_c0_g1	AP-2 complex subunit alpha-1 [*H. sapiens*]	AP2 complex subunit alpha-1 [XP_035253663.1]/*ap2a1*	-0.89	<0.01	198.7 ± 36.1	512.0 ± 16.2	Adaptor – in mammals [118]
DN3510_c0_g1	AP-2 complex subunit alpha-1 [*H. sapiens*]	AP2 complex subunit alpha-1 [XP_035260558.1]/LOC118220647	-0.66	<0.01	5812.5 ± 428.2	9166.3 ± 159.8	Adaptor – in mammals [118]
DN9246_c0_g1	ARHb [*X. laevis*]	Arhb-like [XP_035284714.1]/LOC118233288	-0.08	0.40	12620.8 ± 460.0	12185.0 ± 292.6	Adaptor – in *Xenopus laevis* [88, 89]
DN9305_c0_g1	ARHa [*X. laevis*]	Arhb [XP_035244913.1]/*ldlrap1b*	0.12	0.73	237.5 ± 11.7	343.5 ± 49.6	Adaptor – in *Xenopus laevis* [88, 89]
DN3816_c0_g1	DAB2 [*H. sapiens*]	Dab2 [XP_035234797.1]/si:ch211-204c21.1	-0.78	0.01	4120.23 ± 758.8	7146.8 ± 198.3	Adaptor – in mammals [119]
DN3142_c0_g1	DAB2 [*R. norvegicus*]	Dab2-like [XP_035246863.1]/LOC118212748	-0.48	<0.01	7030.2 ± 628.4	9520.3 ± 256.7	Adaptor – in mammals [119]
DN434_c0_g1	dynamin-2 [*B. taurus*]	dynamin 2-like [XP_035262234.1]/LOC118221351	-0.43	<0.01	1250.9 ± 127.1	2067.1 ± 89.2	Clathrin coated pit scission – in mammals [120], in *Danio rerio* [121]

DN14218_c0_g1	auxilin [*H. sapiens*]	auxilin [XP_035272232.1]/*dnajc6*	-0.86	0.01	47.8 ± 3.5	135.0 ± 14.9	Vesicle uncoating – in mammals [122]
DN5059_c0_g1	RIN2 [*M. musculus*]	Rin1a [XP_035289419.1]/*rin1a*	-0.86	0.13	418.3 ± 101.5	975.2 ± 152.6	Facilitates Ras-activated receptor endocytosis [123]
DN576_c0_g1	RAB1A [*R. norvegicus*]	ORAB-1-like [XP_035256992.1]/LOC118218450	-0.24	0.05	1641.8 ± 152.9	2336.2 ± 73.0	Early endosome – in mammals [112]
DN2384_c0_g1	RAB5A [*M. musculus*]	Rab5aa[XP_035284993.1]/*rab5aa*	-0.26	0.14	275.4 ± 33.0	478.9 ± 12.3	Early endosome [124]
DN754_c0_g1	RAB5A [*H. sapiens*]	Rab5ab [XP_035236399.1]/*rab5ab*	<0.01	0.99	619.0 ± 45.2	831.1 ± 30.9	Early endosome [124]
DN22213_c0_g1	Rab4a [*D. rerio*]	Rab4a [XP_035262813.1]/*rab4a*	0.42	<0.01	796.7 ± 15.3	799.7 ± 30.7	Early endosome [124]
DN432_c0_g1	RAB35 [*R. norvegicus*]	Rab35 [XP_035254099.1]/si:dkey-16l2.16	-0.34	0.04	722.3 ± 97.6	1192.0 ± 55.3	Recycling [125]
DN618_c0_g1	RAB35 [*R. norvegicus*]	Rab35b [XP_035236038.1]/*rab35b*	-0.04	0.75	1283.2 ± 105.2	1644.0 ±70.4	Recycling [125]
DN3244_c0_g1	cathepsin B [*M. fascicularis*]	cathepsin B [XP_035279133.1]/*ctsba*	-0.33	0.05	34962.5 ± 2002.9	34068.2 ± 1206.2	Proteolysis – review on oviparity with focus on teleost fish [94]
DN10366_c0_g1	cathepsin S [*H. sapiens*]	cathepsin S-like [XP_035260247.1]/LOC118220535	0.26	0.11	109445.4 ± 12625.2	58755.7 ± 2018.3	Proteolysis – in *F. heteroclitus* [113]
DN4408_c0_g2	cathepsin C [*P. abelii*]	cathepsin C [XP_035241487.1]/*ctsc*	-0.03	0.84	1462.8 ± 96.7	1834.7 ± 85.6	Proteolysis – in *F. heteroclitus* [113]
DN85011_c0_g1	cathepsin F [*M. musculus*]	cathepsin F [XP_035264650.1]/*ctsf*	0.57	0.05	1305.8 ± 101.0	1143.7 ± 114.9	Proteolysis – in *F. heteroclitus* [113]
DN17646_c0_g1	cathepsin Z [*M. musculus*]	cathepsin Z [XP_035283856.1]/LOC118232826	-0.33	0.26	63.6 ± 5.7	124.7 ± 17.3	Proteolysis – in *F. heteroclitus* [113]

DN48_c0_g1	cathepsin D [*C. harengus*]	cathepsin D [XP_035251717.1]/*ctsd*	0.21	0.660	1601.0 ± 162.8	1889.2 ± 380.6	Proteolysis – review on oviparity with focus on teleost fish [94]

References

97. Miller, S.E.; Mathiasen, S.; Bright, N.A.; Pierre, F.; Kelly, B.T.; Kladt, N.; Schauss, A.; Merrifield, C.J.; Stamou, D.; Höning, S.; Owen, D.J. CALM regulates clathrin-coated vesicle size and maturation by directly sensing and driving membrane curvature. *Dev. Cell* **2015**, *33*, pp. 163–175, https://doi.org/10.1016/j.devcel.2015.03.002
98. Santolini, E.; Puri, C.; Salcini, A.E.; Gagliani, M.C.; Pelicci, P.G.; Tacchetti, C.; Di Fiore, P.P. Numb is an endocytic protein. *The J. Cell Biol.* **2000**, *151*, pp. 1345–1352, https://doi.org/10.1083/jcb.151.6.1345
99. Piccini, A.; Castroflorio, E.; Valente, P.; Guarnieri, F.C.; Aprile, D.; Michetti, C.; Bramini, M.; Giansante, G.; Pinto, B.; Savardi, A.; Cesca, F.; Bachi, A.; Cattaneo, A.; Wren, J.D.; Fassio, A.; Valtorta, F.; Benfenati, F.; Giovedì, S. APache is an AP2-interacting protein involved in synaptic vesicle trafficking and neuronal development. *Cell Rep.* **2017**, *21*, pp. 3596–3611, https://doi.org/10.1016/j.celrep.2017.11.073.
100. Sen, A.; Madhivanan, K.; Mukherjee, D.; Aguilar, R.C. The epsin protein family: coordinators of endocytosis and signaling. *Biomol. Concepts* **2012**, *3*, pp. 117–126, https://doi.org/10.1515/bmc-2011-0060
101. Conner, S.D.; Schröter, T.; Schmid, S.L. AAK1-mediated μ2 phosphorylation is stimulated by assembled clathrin. *Traffic* **2003**, *4*, pp. 885–890, https://doi.org/10.1046/j.1398-9219.2003.0142.x
102. Sorensen, E.B.; Conner, S.D. AAK1 regulates Numb function at an early step in clathrin-mediated endocytosis. *Traffic* **2008**, *9*, pp. 1791–1800, https://doi.org/10.1111/j.1600-0854.2008.00790.x
103. Engqvist-Goldstein, A.E.; Warren, R.A.; Kessels, M.M.; Keen, J.H.; Heuser, J.; Drubin, D.G. The actin-binding protein Hip1R associates with clathrin during early stages of endocytosis and promotes clathrin assembly *in vitro*. *J. Cell Biol.* **2001**, *154*, pp. 1209–1223, https://doi.org/10.1083/jcb.200106089
104. Bai, J.; Hu, Z.; Dittman, J.S.; Pym, E.C.; Kaplan, J.M. Endophilin functions as a membrane-bending molecule and is delivered to endocytic zones by exocytosis. *Cell* **2010**, *143*, pp. 430–441, https://doi.org/10.1016/j.cell.2010.09.024
105. Milosevic, I.; Giovedi, S.; Lou, X.; Raimondi, A.; Collesi, C.; Shen, H.; Paradise, S.; O'Toole, E.; Ferguson, S.; Cremona, O.; De Camilli, P. Recruitment of endophilin to clathrin-coated pit necks is required for efficient vesicle uncoating after fission. *Neuron* **2011**, *72*, pp. 587–601, https://doi.org/10.1016/j.neuron.2011.08.029
106. Park, J.; Kim, Y.; Lee, S.; Park, J.J.; Park, Z.Y.; Sun, W.; Kim, H.; Chang, S. SNX18 shares a redundant role with SNX9 and modulates endocytic trafficking at the plasma membrane. *J. Cell Sci.* **2010**, *123*, pp. 1742–1750, https://doi.org/10.1242/jcs.064170
107. Stockinger, W.; Sailler, B.; Strasser, V.; Recheis, B.; Fasching, D.; Kahr, L.; Schneider, W.J.; Nimpf, J. The PX-domain protein SNX17 interacts with members of the LDL receptor family and modulates endocytosis of the LDL receptor. *EMBO J.* **2002**, *21*, pp. 4259–4267, https://doi.org/10.1093/emboj/cdf435
108. Burden, J.J.; Sun, X.M.; García, A.B.G.; Soutar, A.K. Sorting motifs in the intracellular domain of the low density lipoprotein receptor interact with a novel domain of sorting nexin-17. *J. Biol. Chem.* **2004**, *279*, pp. 16237–16245, https://doi.org/10.1074/jbc.M313689200
109. Van Kerkhof, P.; Lee, J.; McCormick, L.; Tetrault, E.; Lu, W.; Schoenfish, M.; Oorschot, V.; Strous, G.J.; Klumperman, J.; Bu, G. Sorting nexin 17 facilitates LRP recycling in the early endosome. *EMBO J.* **2005**, *24*, pp. 2851–2861, https://doi.org/10.1038/sj.emboj.7600756
110. Saito, K.; Murai, J.; Kajiho, H.; Kontani, K.; Kurosu, H.; Katada, T. A novel binding protein composed of homophilic tetramer exhibits unique properties for the small GTPase Rab5. *J. Biol. Chem.* **2002**, *277*, pp. 3412–3418, https://doi.org/10.1074/jbc.M106276200
111. Gournier, H.; Stenmark, H.; Rybin, V.; Lippe, R.; Zerial, M. Two distinct effectors of the small GTPase Rab5 cooperate in endocytic membrane fusion. *EMBO J.* **1998**, *17*, pp. 1930–1940, https://doi.org/10.1093/emboj/17.7.1930

112. Mukhopadhyay, A.; Quiroz, J.A.; Wolkoff, A.W. Rab1a regulates sorting of early endocytic vesicles. *Am. J. Physiol. Gastrointest. Liver Physiol.* **2014**, *306*, G412–G424, https://doi.org/10.1152/ajpgi.00118.2013
113. Fabra, M.; Cerdà, J. Ovarian cysteine proteinases in the teleost *Fundulus heteroclitus*: molecular cloning and gene expression during vitellogenesis and oocyte maturation. *Mol. Reprod. Dev. Gamete Res.* **2004**, *67*, pp. 282–294, doi: 10.1002/mrd.20018.
114. Riggio, M.; Scudiero, R.; Filosa, S.; Parisi, E. Sex-and tissue-specific expression of aspartic proteinases in *Danio rerio* (zebrafish). *Gene* **2000**, *260*, pp. 67–75, https://doi.org/10.1016/s0378-1119(00)00469-8
115. Hiramatsu, N.; Luo, W.; Reading, B.J.; Sullivan, C.V.; Mizuta, H.; Ryu, Y.W.; Nishimiya, O.; Todo, T.; Hara, A. Multiple ovarian lipoprotein receptors in teleosts. *Fish Physiol. Biochem.* **2013**, *39*, pp. 29–32, https://doi.org/10.1007/s10695-012-9612-6
116. Royle, S.J. The cellular functions of clathrin. *Cell. Mol. Life Sci.* **2006**, *63*, pp. 1823–1832, https://doi.org/10.1007/s00018-005-5587-0
117. Mizuta, H.; Mushirobira, Y.; Nagata, J.; Todo, T.; Hara, A.; Reading, B.J.; Sullivan, C.V.; Hiramatsu, N. Ovarian expression and localization of clathrin (Cltc) components in cutthroat trout, *Oncorhynchus clarki*: evidence for Cltc involvement in endocytosis of vitellogenin during oocyte growth. *Comp. Biochem. Physiol. Part A Mol. Integr. Physiol.* **2017**, *212*, pp. 24–34, https://doi.org/10.1016/j.cbpa.2017.06.021
118. Kelly, B.T.; Graham, S.C.; Liska, N.; Dannhauser, P.N.; Honing, S.; Ungewickell, E.J.; Owen, D.J. AP2 control clathrin polymerization with a membrane-activated switch. *Science* **2014**, *345*, pp. 459–463, https://doi.org/10.1126/science.1254836
119. Maurer, M.E.; Cooper, J.A. The adaptor protein Dab2 sorts LDL receptors into coated pits independently of AP-2 and ARH. *J. Cell Sci.* **2006**, *119*, pp. 4235–4246, https://doi.org/10.1242/jcs.03217
120. Merrifield, C.J.; Feldman, M.E.; Wan, L.; Almers, W. Imaging actin and dynamin recruitment during invagination of single clathrin-coated pits. *Nat. Cell Biol.* **2002**, *4*, pp. 691–698, https://doi.org/10.1038/ncb837
121. Gibbs, E.M.; Davidson, A.E.; Trickey-Glassman A.; Backus C.; Hong Y.; Sakowski, S.A.; Dowling, J.J.; Feldman, E.L. Two dynamin-2 genes are required for normal zebrafish development. *PLoS ONE* **2013**, *8*, e55888, https://doi.org/10.1371/journal.pone.0055888
122. Massol, R.H.; Boll, W.; Griffin, A.M.; Kirchhausen, T. A burst of auxilin recruitment determines the onset of clathrin-coated vesicle uncoating. *Proc. Natl. Acad. Sci.* **2006**, *103*, pp. 10265–10270, https://doi.org/10.1073/pnas.0603369103
123. Tall, G.G.; Barbieri, M.A.; Stahl, P.D.; Horazdovsky, B.F. Ras-activated endocytosis is mediated by the Rab5 guanine nucleotide exchange activity of RIN1. *Dev. Cell* **2001,** *1*, pp. 73-82, https://doi.org/10.1016/s1534-5807(01)00008-9
124. Wandinger-Ness, A.; Zerial, M. Rab proteins and the compartmentalization of the endosomal system. *Cold Spring Harb. Perspect. Biol.* **2014**, *6*, a022616, https://doi.org/10.1101/cshperspect.a022616
125. Grant, B.D.; Donaldson, J.G. Pathways and mechanisms of endocytic recycling. *Nat. Rev. Mol. Cell Biol.* **2009**, *10*, pp. 597–608, https://doi.org/10.1038/nrm2755
